# Adjuvant Effect of Orally Applied Preparations Containing Non-Digestible Polysaccharides on Influenza Vaccination in Healthy Seniors: A Double-Blind, Randomised, Controlled Pilot Trial

**DOI:** 10.3390/nu13082683

**Published:** 2021-08-02

**Authors:** Christiane Laue, Yala Stevens, Monique van Erp, Ekaterina Papazova, Edlyn Soeth, Angelika Pannenbeckers, Ellen Stolte, Ruwen Böhm, Sophie Le Gall, Xavier Falourd, Simon Ballance, Svein H. Knutsen, Iris Pinheiro, Sam Possemiers, Paul M. Ryan, R. Paul Ross, Catherine Stanton, Jerry M. Wells, Sylvie van der Werf, Jurriaan J. Mes, Juergen Schrezenmeir

**Affiliations:** 1 Clinical Research Center Kiel, Kiel Center of Innovation and Technology, 24118 Kiel, Germany; e.papazova@crc-kiel.de (E.P.); edlyn.soeth@uksh.de (E.S.); a.pannenbeckers@crc-kiel.de (A.P.); ruwen.boehm@pharmakologie.uni-kiel.de (R.B.); j.schrezenmeir@crc-kiel.de (J.S.); 2 BioActor, Brightlands Health Campus, 6229 GS Maastricht, The Netherlands; yala.stevens@bioactor.com (Y.S.); monique.bioactor@gmail.com (M.v.E.); 3 Host-Microbe Interactomics, Wageningen University & Research, 6708 WD Wageningen, The Netherlands; ellen.kranenbarg@wur.nl (E.S.); jerry.wells@wur.nl (J.M.W.); 4 UR1268 BIA, INRA, 44316 Nantes, France; sophie.le-gall@inrae.fr (S.L.G.); xavier.falourd@inrae.fr (X.F.); 5 Nofima, Norwegian Institute of Food Fisheries & Aquaculture Research , 1433 Ås, Norway; simon.balance@nofima.no (S.B.); svein.knutsen@nofima.no (S.H.K.); 6 Prodigest, Technologiepark-Zwijnaarde, 9052 Ghent, Belgium; iris.pinheiro@mrmhealth.com (I.P.); sam.possemiers@mrmhealth.com (S.P.); 7 Teagasc, Food Research Centre, Moorepark, Fermoy, Co., P61 C996 Cork, Ireland; paul_ryan@umail.ucc.ie (P.M.R.); catherine.stanton@teagasc.ie (C.S.); 8 APC Microbiome Ireland, University College Cork, T12 YT20 Cork, Ireland; p.ross@ucc.ie; 9 Institut Pasteur, 75015 Paris, France; sylvie.van-der-werf@pasteur.fr; 10 Wageningen Food and Biobased Research, Wageningen University & Research, 6708 WG Wageningen, The Netherlands; jurriaan.mes@wur.nl

**Keywords:** adjuvant, immunity, non-digestible polysaccharide, prebiotics, arabinoxylan, β-glucan, cold, influenza, vaccination

## Abstract

Senior individuals can suffer from immunosenescence and novel strategies to bolster the immune response could contribute to healthy ageing. In this double-blind, randomised, controlled pilot trial, we investigated the ability of non-digestible polysaccharide (NPS) preparations to enhance the immune response in a human vaccination model. In total, 239 subjects (aged 50–79 years) were randomised to consume one of five different NPS (yeast β-glucan (YBG), shiitake β-glucan (SBG), oat β-glucan (OBG), arabinoxylan (AX), bacterial exopolysaccharide (EPS)) or control (CTRL) product daily for five weeks. After two weeks of intervention, subjects were vaccinated with seasonal influenza vaccine. The post-vaccination increases in haemagglutination inhibition antibody titres and seroprotection rate against the influenza strains were non-significantly enhanced in the NPS intervention groups compared to CTRL. Specifically, a trend towards a higher mean log_2_ fold increase was observed in the AX group (uncorrected *p* = 0.074) combined with a trend for an increased seroprotection rate, AX group (48.7%) compared to CTRL (25.6%) (uncorrected *p* = 0.057), for the influenza A H1N1 strain. Subjects consuming AX also had a reduced incidence of common colds compared to CTRL (1 vs. 8; *p* = 0.029 in Fisher exact test). No adverse effects of NPS consumption were reported. The findings of this pilot study warrant further research to study AX as an oral adjuvant to support vaccine efficacy.

## 1. Introduction

The process of ageing is associated with a deterioration in the function of the immune system, referred to as immunosenescence [1]. As a consequence of impaired immune responses, the elderly are highly susceptible to infection and have an increased risk of complications resulting in hospitalisation and mortality [1,2]. Although vaccination is considered the most effective measure to prevent or reduce the severity of infections and their associated complications [2], the response to vaccination in the elderly has been found to be considerably weaker than in younger adults, due to immunosenescence [3]. To improve protection against infectious diseases in the elderly, there is a need for novel strategies to boost the immune response [4]. Nutritional interventions might be promising strategies to modulate immune system responsiveness, thereby improving health and reducing disease risk [5].

To assess the immunomodulatory effects of foods or food components, a human vaccination model is considered highly suitable [5]. In this model, stimulation of the immune response to a standard vaccination (e.g., seasonal influenza vaccination), which can be measured by increased vaccine-specific serum antibodies, indicates immunostimulatory effects and enhancement of immune defences. Additionally, according to the European Food Safety Authority (EFSA), stimulation of vaccination responses, as measured by increased numbers of individuals attaining protective antibody levels or by increments in antibody titres in groups of individuals, is an appropriate outcome for the scientific substantiation of beneficial effects of food components on the function of the immune system related to immune defence against pathogens [6,7]. Therefore, novel nutritional strategies targeting the immune response should be investigated in human vaccination trials to provide conclusive evidence on their beneficial immunomodulatory effects.

Non-digestible polysaccharides (NPS) are increasingly recognised for their potential immunostimulatory properties. There is strong evidence for direct and indirect beneficial effects of NPS intake on the immune response [8,9]. The NPS carbohydrate structures themselves can exert direct immunomodulatory effects by interaction with pattern recognition receptors on the surface of immune cells [10]. In addition, indirect beneficial effects of NPS on the immune system may be exerted through modulation of the intestinal microbiota and the production of metabolites such as short-chain fatty acids (SCFA) [9,11], which are considered important regulators of the immune response [12]. Indeed, many fermentable NPS are considered candidate prebiotics, beneficially affecting host health by selectively stimulating the growth and/or activity of a limited number of health-promoting bacterial species resident in the intestinal colon [13]. Given their immunomodulatory properties, a number of polysaccharide compounds from plants, bacteria, fungi, and synthetic sources have emerged as promising vaccine adjuvant candidates [10,14]. Among these polysaccharides, the NPS β-glucans, arabinoxylans and exopolysaccharides are of particular interest, as their immunomodulatory properties have been studied with promising results in vitro as well as in vivo [15,16,17,18,19,20,21,22,23].

β-Glucans represent a heterogeneous group of homopolysaccharides composed of β-linked D-glucose residues that are found in cereal grains (e.g., oat, barley), in the cell wall of *Saccharomyces cerevisiae* (baker’s yeast) and in different edible mushrooms and seaweed [24]. In humans, a particular β-glucan from *S. cerevisiae* was found to reduce the number of influenza symptoms and duration and severity of upper respiratory tract infections in three randomised, placebo-controlled trials [25,26,27]. Furthermore, studies in mice have shown that lentinan, a β-glucan from shiitake (*Lentinula edodes*), could enhance vaccine efficacy [28,29].

Arabinoxylans (AX) are plant cell wall heteropolysaccharides consisting of a linear β-linked xylose backbone with variable side chains of arabinose and/or other sugar residues and different degrees of polymerization, depending on their origin in the grain [30]. AX is the most abundant dietary fibre in cereal grain endosperm of wheat and rye and has been shown to significantly increase the antibody response to sheep red blood cell injection in chicken, indicating its potential to stimulate antibody mediated immune responses [31].

Exopolysaccharides (EPS) are extracellular polysaccharides secreted by lactic acid bacteria and consist of branched, repeating units of sugars with variable structures and compositions [32]. Several EPS have been reported to possess immunomodulatory or potential adjuvant activities to vaccination in vitro and in vivo [22,23,33,34]. Indeed, EPS from the strain utilised in the present study, *Limosilactobacillus* *mucosae* DPC 6426, has previously been shown to be highly immunomodulatory in macrophages under in vitro conditions [35]. Despite the emerging evidence suggesting that NPS may enhance the immune response to vaccination, the effectiveness of NPS as oral vaccine adjuvants has not been confirmed yet in humans [4]. Therefore, the aim of this pilot trial was to investigate the effects of a five-week consumption period of different dietary NPS on the antibody response to influenza vaccination, respiratory tract infections and cellular immunity of healthy volunteers aged 50–79 years. Furthermore, the effects on faecal microbiota and its metabolites as well as on gastrointestinal wellbeing were investigated.

## 2. Materials and Methods

The clinical trial was approved by the ethics committee (Ärztekammer Schleswig-Holstein, Ethik-Kommission, DE/EKSH44) on 8 August 2012 and was prospectively registered at ClinicalTrials.gov (NCT01896154). The trial was conducted at Clinical Research Center Kiel (Kiel, Germany) in the period from 28 August 2012 to 27 March 2013. All subjects gave written informed consent for participation in the study.

### 2.1. Study Population

Eligible subjects were community-dwelling men and postmenopausal women, aged 50–79 years, willing to have an influenza vaccination in season 2012/2013. Subjects were recruited from the database of Clinical Research Center Kiel and from advertisements in the Kieler daily newspaper. Among other conditions, suffering from influenza or influenza-like illness within the previous 10 months and usage of drugs altering the immune system (e.g., antibiotics or corticosteroids) led to exclusion of subjects. The full list of eligibility criteria is available at ClinicalTrials.gov and in the supplement (Methods S1).

### 2.2. Study Products

Subjects were asked to consume one sachet of NPS powder or maltodextrin (Glucidex IT, Roquette Frères, Lestrem, France; 12.0 g, control (CTRL)), stirred in 200 mL milk or apple juice once daily for five weeks. The sachets with NPS powder contained either: (i) a β-glucan preparation from yeast (Wellmune^®^, Soluble Powder, Lot 12111-016, Biothera, Eagan, MN, USA supplied by Immitec, Nøtterøy, Norway; 500 mg, YBG), (ii) a β-glucan preparation from shiitake prepared according to a pre-specified procedure (Methods S2) by Wageningen Food and Biobased Research (Wageningen, the Netherlands; 500 mg, SBG), (iii) a β-glucan preparation from oat (Oatwell^®^ 28%, Swedish Oat Fiber AB, Bua, Sweden; 10.0 g, OBG), (iv) an arabinoxylan preparation from wheat endosperm (Naxus^®^, BioActor, Maastricht, the Netherlands; 10.0 g, AX) or (v) an exopolysaccharide preparation from *Limosilactobacillus mucosae* DPC 6426 prepared according to a detailed procedure (Methods S3) by Teagasc (Fermoy, Ireland; 2.3 g, EPS). More detailed information on the NPS powders can be found in Supplementary Table S1 and was slightly modified from [36]. All sachets with NPS powder were supplemented with maltodextrin adding up to a net weight of 12.0 g. Before and after mixing into milk or apple juice, the NPS test products and CTRL product had a similar appearance and taste.

### 2.3. Study Design

This study was designed as a randomised, controlled, double-blind, parallel-group study. Subjects were randomly assigned to one of six intervention groups: YBG, SBG, OBG, AX, EPS, or CTRL. To avoid selection bias, a randomisation list was generated by data managers of tecura GmbH (Kiel, Germany) in line with the Cochrane guidelines [37] using a software implementation by G.E. Dallal of the pseudo-random number generator of Wichmann and Hill [38] (http://www.randomization.com(accessed on 17 July 2012)). The randomisation list was kept confidential at the premises of tecura GmbH and was only provided to Nofima (Ås, Norway) for filling sachets with the intervention products and labelling. Nofima was solely responsible for this task and not involved in the study conduct. The key for unblinding was maintained until statistical analyses were performed by statistical managers, who were not otherwise involved in conducting the study. Neither the investigators nor the subjects were aware of the content of the sachets until all analyses were completed. 

**Figure 1:**
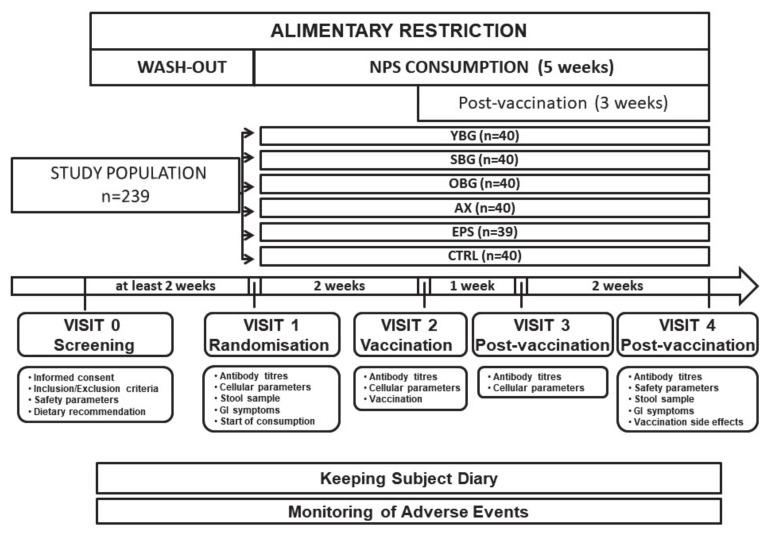
Design of the pilot study (*n* = 239). A screening visit (visit 0) was conducted at least two weeks prior to randomisation (visit 1). After randomisation, subjects started consuming NPS or CTRL product daily for five weeks. After two weeks consumption, subjects were vaccinated against influenza (visit 2). Follow-up visits were conducted one week (visit 3) and three weeks (visit 4) post-vaccination. Black boxes indicate parameters that were measured at each visit. Subjects kept a diary assessing compliance and respiratory symptoms and followed a low dietary fibre diet for the complete duration of the study. Adverse events were monitored by investigators throughout the study. NPS: non-digestible polysaccharide, YBG: yeast β-glucan, SBG: shiitake β-glucan, OBG: oat β-glucan, AX: arabinoxylan, EPS: exopolysaccharide, CTRL: control (maltodextrin), GI: gastrointestinal.

The study lasted at least seven weeks per subject with a two-week period of wash-out between enrolment (visit 0 (V0)) and randomisation (V1), followed by two weeks of test product consumption once daily until vaccination (V2) and continued product consumption for three weeks post-vaccination with visits after one week (V3) and after two additional weeks (V4) (Figure 1). Subjects were instructed to abstain from foods and supplements containing probiotics, prebiotics or other fermented products, supplements containing vitamins and minerals as well as to sustain a low dietary fibre diet for the complete duration of the study (V0–V4). This was based on the notion that these dietary factors may exert immunomodulatory effects and aimed at keeping background noise by interfering factors low. Compliance and adverse events were monitored and recorded by the investigators throughout the study.

### 2.4. Compliance Assessment

Subjects were asked to return any unused test products at V4, which were counted in order to calculate compliance (the number of test products consumed related to the target number of test products to be consumed (consumed/target × 100%)). Moreover, compliance was assessed by the Morisky score. Subjects completed a questionnaire concerning product consumption compliance by answering four questions according to Morisky et al. [39]. Compliance was defined as low if three or four questions were answered with “yes”, medium if one or two questions were answered with “yes”, and high if none of the questions were answered with “yes”.

### 2.5. Influenza Vaccine

Subjects were vaccinated at V2 with the split virion, inactivated influenza vaccine Vaxigrip^®^ (season 2012/2013; Sanofi-Pasteur MSD, Lyon, France), containing an A/California/7/2009 (H1N1)pdm09-like virus, an A/Victoria/361/2011 (H3N2)-like virus and a B/Wisconsin/1/2010-like virus (Influenza B). This vaccine complied with the WHO recommendations for the Northern Hemisphere and EU decision for the 2012/2013 influenza season [40].

### 2.6. Acquisition of Blood

Blood was drawn from the median cubital vein in the antecubital fossa using a 21 G butterfly needle and collected with the blood collection system S-Monovette^®^ (Sarstedt, Nümbrecht, Germany). For blood cell counts EDTA monovettes, for electrolytes, liver enzymes, creatinine and haemagglutination inhibition (HI) titres serum monovettes with clot activator and for whole blood incubation (cellular immunity) lithium-heparin monovettes were used.

### 2.7. Antibody Titres

Influenza-specific serum antibody titres against each of three influenza strains (H1N1, H3N2, and Influenza B) were quantified by a standard HI assay as recommended by the European Medicines Agency (EMA) [41]. HI titres against each of the influenza strains were measured in duplicate in 0.5 titre steps, and their geometric mean titre (GMT) was transformed using log_2_(titre/10). Log-transformed titres were provided in 0.25 titre steps. These GMT were used to calculate mean log_2_ fold increase (MLFI) between baseline (V1) and three weeks post-vaccination (V4) (Log_2_(titre V4/titre V1)). In addition, seroprotection and seroconversion rates were calculated. The seroprotection rate is defined as the percentage of subjects attaining an antibody titre ≥40 in the HI assay. Seroconversion rate is defined as the percentage of subjects with an HI antibody titre <10 at baseline and a post-vaccination titre ≥40 or a titre ≥10 at baseline and at least a fourfold increase in titre post-vaccination [42].

In addition to HI assays, micro-neutralization assays were performed. After heat inactivation at 56 °C for 30 min, serial two-fold dilutions of serum (1:10 to 1:320) were added in quadruplicate to 10^3^ TCID_50_ of the influenza strains and incubated at 37 °C for two hours before being transferred to a 96-well microtiter plate containing confluent MDCK cells. Plates were incubated for three days at 37 °C under a humidified atmosphere containing 5% CO_2_. The neutralization antibody titre, expressed as the reciprocal of the highest serum dilution providing complete protection from infection, was used to calculate the GMT of quadruplicates, MLFI between baseline (V1) and three weeks post-vaccination (V4), and seroprotection and seroconversion rates.

### 2.8. Respiratory Tract Infections

During the intervention period, respiratory tract infection (RTI) symptoms were assessed at V1, V2, V3, and V4.

#### 2.8.1. Common Cold (Upper Respiratory Tract Infection)

The severity of common cold episodes (with rhinitis, pharyngitis, and general signs) was quantified during the intervention period (V1–V4) by the total symptom score, i.e., the sum of the daily symptom scores according to Predy et al. [43] across all days of a common cold episode during the study period. These scores were based on 10 symptoms (runny nose, sneeze, nasal congestion, sore throat, hoarseness, cough, malaise, fever, headache, and earaches) rated on a four-point scale (0 = no symptoms, 1 = mild symptoms, 2 = moderate symptoms, and 3 = severe symptoms). A two-day total symptom score greater than 14 was considered to indicate a verified cold; these were used in the analysis of number of colds. A daily symptom score exceeding 4 defined the days with symptoms of cold and by that defined the duration of cold episodes.

A further evaluation was based on common cold criteria according to Jackson et al. [44]. According to these criteria, the daily symptom score was based on seven symptoms (sneezing, nasal discharge, nasal obstruction, sore throat, cough, headache, and malaise), the degree of which was estimated on a four-point scale (0 = no symptoms, 1 = mild symptoms, 2 = moderate symptoms, and 3 = severe symptoms). This was one symptom (i.e., chilliness) less than the original eight-symptom score of Jackson. A common cold was verified if two out of three listed criteria were met: (1) a total symptom score of 14 or more over baseline during a 6-day period, (2) the impression of the subject that a common cold had developed, (3) an increase in nasal discharge on three or more out of six days.

#### 2.8.2. Influenza or Influenza-Like Illness

Influenza-like illness was evaluated according to the Centre for Disease Control and Prevention criteria (CDC, Atlanta, GA, USA). It was defined by a body temperature ≥37.8 °C and concomitantly either cough or sore throat. In addition to the CDC criteria, the time course of onset of the disease was recorded.

#### 2.8.3. Acute Bronchitis and Pneumonia (Lower Respiratory Tract Infection)

Acute bronchitis and pneumonia were diagnosed by the following clinical symptoms: common cold symptoms, expectorations, retrosternal burning and—only in the case of pneumonia—dyspnoea, tachypnoea, tachycardia, and inspiratory rales.

### 2.9. Cellular Immunity

Within 60 min after blood withdrawal at V1, V2, and V3, fresh heparinised whole blood of subjects was diluted five times with RPMI1640 medium (Invitrogen^®^, Thermo Fisher Scientific, Waltham, MA, USA) containing 100 units/mL penicillin and 100 µg/mL streptomycin. Diluted blood (950 µL, each) was added to 48-well plates containing 50 µL medium or 100 ng/mL lipopolysaccharides (LPS, *E.coli* LPS ultra-pure, Cayla-InvivoGen, Toulouse, France) or 5 µg/mL of the lectin concanavalin A (ConA, Concavalin A Type IV, Sigma Aldrich, St. Louis, MO, USA) as stimulants and incubated for 24 h at 37 °C and 5% CO_2_. After 24 h, cell culture supernatants were harvested into 96-well plates and stored at −20 °C until cytokine analysis. Cytokines of the innate and cellular immune response (interferon-γ (IFN-γ), tumour necrosis factor α (TNF-α), interleukin (IL)-1β, IL-2, IL-12, and IL-10) were measured in the supernatants using Bio-Plex Pro Reagent Kit (Bio-Rad, Veenendaal, the Netherlands) according to the manufacturer’s instructions. In brief, 50 μL magnetic beads were dispersed in a plate together with 50 μL of supernatant or standard and incubated for 30 min at room temperature while shaking 300 rpm in the dark. Subsequently, the beads were incubated with 25 μL detection antibodies for 30 min at room temperature while shaking 300 rpm in the dark followed by 50 μL streptavidin-PE for 10 min at room temperature while shaking 300 rpm in the dark. The beads were resuspended in 125 μL assay buffer and read by Bio-Plex^®^ MAGPIX™ Multiplex Reader (Bio-Rad). Data processing was performed using Bio-Plex Manager 5.0, and concentrations (in pg/mL) were interpolated from standard curves. In the few cases that data points were below or above the standard curves, the lower or upper limits of the standard curve were used for data points, respectively.

### 2.10. Microbiota Analysis

Subjects were instructed to collect five stool samples at home the first day of defecation within the last three days prior to the test visit, to freeze them at −20 °C and bring them to the study site at V1 and V4. Faecal microbiota composition after five weeks intervention (V4) were analysed solely for OBG and AX compared to CTRL, as these two intervention groups contained the highest dosage of NPS. 

Sequencing of microbial rRNA samples was performed at Teagasc (Fermoy, Ireland). The V3-V4 regions of the 16S gene were amplified using the primer pair 5′-TCGTCGGCAGCGTCAGATGTGTATAAGAGACAGCCTACGGGNGGCWGCAG-3′ and 5′-GTCTCGTGGGCTCGGAGATGTGTATAAGAGACAGGACTACHVGGGTATCTAATCC-3′, as per the preparation instructions for Illumina MiSeq (San Diego, CA, USA). Samples were barcoded using the primer combinations available in the Illumina Nextera XT kit (Illumina) prior to quantification with the Qubit high-sensitivity DNA kit (Life Technologies, Thermo Fisher Scientific, Waltham, MA, USA), equimolar pooling and high-throughput sequencing on the MiSeq platform. Bioinformatical analyses were performed as described elsewhere [45].

### 2.11. Faecal pH and Short-Chain Fatty Acids

Faecal pH and SCFA were analysed for OBG and AX compared to CTRL. To determine the faecal pH, a sample of approximately 0.5 g faeces was homogenised by mixing into 4 mL demineralised water and the pH was immediately measured upon homogenisation. SCFA were analysed as described by De Weirdt et al. [46]. In short, SCFA were extracted from the faecal samples with diethyl ether, after the addition of 2-methyl hexanoic acid as an internal standard. Extracts were analysed using a GC-2014 gas chromatograph (Shimadzu, ‘s-Hertogenbosch, the Netherlands) equipped with a capillary fatty acid-free EC-1000 Econo-Cap column (dimensions: 25 mm × 0.53 mm, film thickness 1.2 μM; Alltech, Laarne, Belgium), a flame ionisation detector, and a split injector. The injection volume was 1 μL and the temperature profile was set from 110 to 160 °C, with a temperature increase of 6 °C/min. The temperature of the injector and detector were 100 and 220 °C, respectively. Nitrogen was used as a carrier gas. Total SCFA were calculated by summing the molar concentrations of acetate, propionate, and butyrate.

### 2.12. Gastrointestinal Symptoms, Stool Consistency and Stool Frequency

Gastrointestinal symptoms and discomfort over the weeks before and during intervention were assessed at V1 and V4, respectively, using the irritable bowel syndrome (IBS) score by Francis [47]. Stool frequency and stool consistency were assessed by the Bristol stool scale [48]. The Bristol score (weighted consistency) was calculated as follows:

(1) $$Bristol\;score=\sum_{i=1}^{7}w_{i}f_{i}$$

where *w_i_* = weighting factor assigned to each stool type of the Bristol stool scale (3, 2, 1, 0, −1, −2, −3 for stool type 1 to type 7) and fi = frequency of stool type (0 = never, 1 = sometimes, 2 = frequently).

### 2.13. Diet

In order to monitor dietary factors that might exert immunomodulatory effects and to assess whether the volunteers were compliant with the request for abstaining from a high-fibre diet, which might interfere with NPS effects, the dietary intake of subjects was analysed using the food frequency questionnaire (FFQ) provided by the German Institute of Human Nutrition Potsdam-Rehbrücke [49] before intervention (V1; diet history with regard to the past 12 months before intervention) and after the intervention period (V4; diet history with regard to the five weeks of intervention).

### 2.14. Safety Laboratory Parameters

Blood cell counts, liver enzymes, electrolytes, and renal function were assessed in an accredited laboratory (Labor Dr. Krause & Kollegen MVZ GmbH, Kiel, Germany) using a clinical laboratory automation system (Cobas^®^ analyzer series, Roche, Basel, Switzerland).

### 2.15. Vaccination Side Effects

Vaccination side effects were recorded and monitored as adverse events. According to the EMA [41], the frequency of local reactions (i.e., indurations >50 mm diameter, ecchymosis) and general symptoms (i.e., temperature ≥38 °C for >24 h, malaise, shivering) were assessed during the total post-vaccination period (V2–V4).

### 2.16. Statistical Analysis

No a priori sample size calculation has been performed since the current study was designed as a pilot study. An initial group size of 40 subjects (total 240 subjects) was chosen. This group size is comparable to the actual sample size used in a pilot trial (*n* = 86, two parallel groups) with a probiotic (*Lactobacillus* spp.) using similar methods as used in this study [50]. Even though the trial was designed as a pilot trial, the geometric mean titre of antibodies against one of the three influenza strains in the HI test was defined a priori as the primary outcome measure. All other parameters were regarded as exploratory.

Normality of the data was evaluated using the Shapiro–Wilk test and the appropriate statistical tests were applied accordingly: one-way ANOVA (parametric) or Kruskal–Wallis one-way ANOVA on ranks followed by Mann–Whitney U pairwise comparisons for differences between groups (non-parametric). Differences in dietary intake over time within the complete study population were tested by Wilcoxon signed rank test. Occurrences were tested using a Chi-square test with post-hoc Fisher exact test. For all outcomes, available case analyses were performed. No data were imputed. 

All statistical analyses were performed using IBM SPSS Statistics for Windows (version 25.0, Armonk, NY, USA) and microbiota results were visualised with Graphpad Prism (version 5.03, San Diego, CA, USA). Normally distributed data are presented as mean ± standard deviation (SD) and medians with interquartile ranges (IQR) for data that were not normally distributed. Two-sided *p*-values ≤ 0.05 are considered significant. If significant, uncorrected *p*-values for post-hoc tests were corrected for multiple testing by the false-discovery rate (FDR) of Benjamini–Hochberg.

## 3. Results

### 3.1. Study Subjects

**Figure 2:**
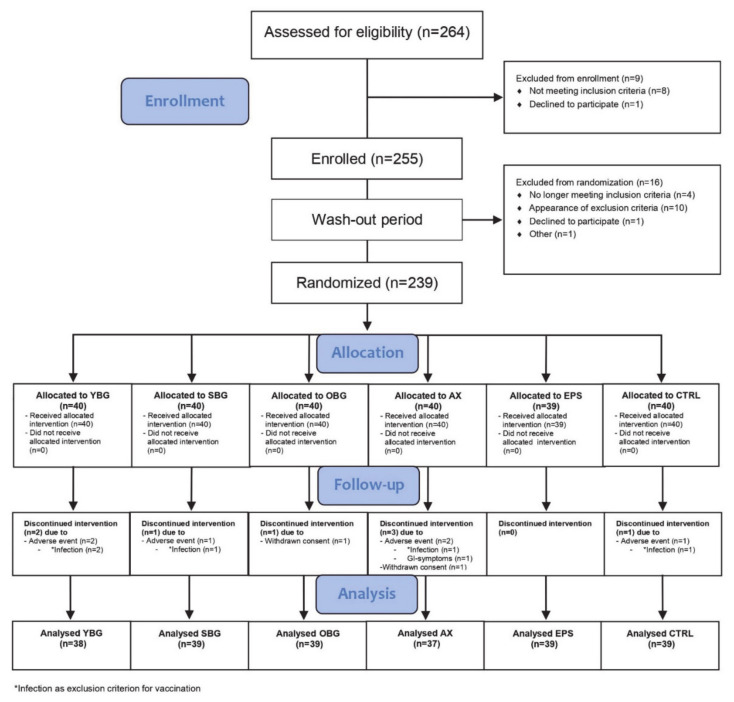
CONSORT flow diagram of the pilot study. YBG: yeast β-glucan, SBG: shiitake β-glucan, OBG: oat β-glucan, AX: arabinoxylan, EPS: exopolysaccharide, CTRL: control.

In this study, a total of 239 subjects aged 50–79 years were included, of which 231 completed the entire study protocol. Five subjects dropped out due to an infection prior to vaccination (common cold, bronchitis, influenza-like illness, urinary tract infection), one subject due to gastrointestinal complaints, and two subjects withdrew consent (Figure 2). Baseline characteristics of the study population are presented in Table 1. The six intervention groups did not differ in sex, age, or BMI. Overall, adherence to the study product consumption was high. The mean compliance by product counting was 98.9%, with 94.6% of the study subjects having a compliance exceeding 95%, which did not differ between intervention groups (*p* = 0.888; data not shown). According to the Morisky score [39], 95.7% of subjects had a *high* compliance, whereas 4.3% had a *medium* compliance. No differences in compliance between intervention groups were observed (*p* = 0.222; data not shown).

### 3.2. Antibody Titres

#### 3.2.1. HI Antibodies

As shown in Table 2, the MLFI for influenza A H1N1 antibody titres was lower in the SBG group compared to CTRL (uncorrected *p* = 0.044), while a trend towards a higher MLFI was observed in the AX group (uncorrected *p* = 0.074) after NPS supplementation. However, these effects were not significant after correction for multiple testing. Furthermore, the increase in the seroprotection rate during the intervention tended to be higher in the AX group (48.7%) compared to the CTRL (25.6%) for the influenza A H1N1 strain (uncorrected *p* = 0.057) (Table 3).

#### 3.2.2. Micro-Neutralization Antibodies

The same analyses as for the HI titres were performed with micro-neutralization titres and showed significant effects on influenza A H1N1 outcomes. In the YBG and SBG groups, the MLFI was lower compared to CTRL (uncorrected *p* = 0.090 and *p* = 0.014, respectively), although this effect did not reach significance for the YBG group (Table 4). The increase in seroprotection rate and seroconversion rate during the intervention period were also lower in the SBG group (30.8% and 39.5%) compared to CTRL (61.5% and 61.5%) for this influenza strain (uncorrected *p* = 0.012 and *p* = 0.069, respectively) (Table 5). After correction for multiple testing, these effects were no longer significant.

### 3.3. Common Cold Incidence

Overall, no significant differences in the occurrence of colds were observed between groups during the intervention period (Table 6). However, in all groups, fewer subjects were affected by colds than in the CTRL group, which was most pronounced in the AX group (one vs. eight colds according to Jackson criteria; uncorrected *p* = 0.029 for Fisher exact test).

### 3.4. Cytokine Production

Cytokine production was measured after incubation of whole blood with culture medium, ConA, or LPS. In the YBG, OBG, AX, and EPS groups, secreted IFN-γ levels in blood incubated with medium changed significantly between baseline and one week after vaccination compared to CTRL (Table S2; all uncorrected and corrected *p* ≤ 0.05). For the other cytokines or treatment conditions, no significant effects were observed over time between groups.

### 3.5. Microbiota Composition

**Figure 3:**
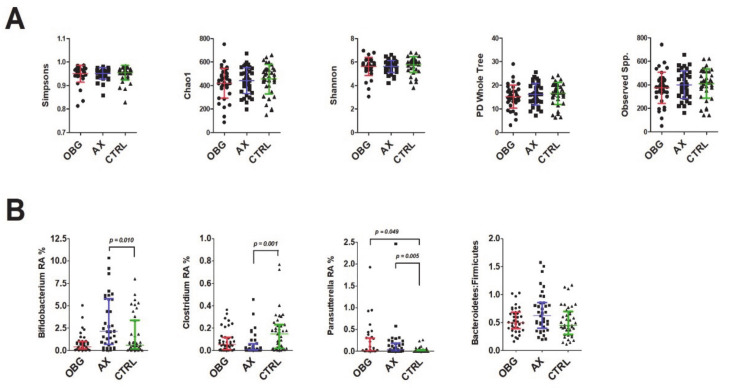
(**A**) α-diversity plots and (**B**) relative abundances (RA) of *Bifidobacterium*, *Clostridium*, *Parasutterella* and Bacteroidetes:Firmicutes ratio in faecal microbiota after five weeks of oat β-glucan (OBG), arabinoxylan (AX) or control (CTRL) consumption. Values are presented as mean ± SD or median ± IQR and differences between intervention groups were tested with one-way ANOVA (**A**) or a Kruskal–Wallis test (**B**), respectively. *p*-values represent significant differences for the pairwise comparisons between OBG or AX compared to CTRL, tested by uncorrected Mann–Whitney U test.

At the end of the five-week consumption period with either AX or OBG, no differences were observed in microbiota α-diversity compared to CTRL (Figure 3A). However, significant differences in the abundance of several bacterial genera were noted. AX-intervened subjects showed a significantly higher relative abundance of *Bifidobacterium* (uncorrected *p* = 0.010), whereas a significantly lower *Clostridium* relative abundance was observed in this group compared to CTRL (uncorrected *p* = 0.001; Figure 3B). A non-significant trend towards a lower *Clostridium* relative abundance was seen for OBG (uncorrected *p* = 0.063). The relative abundance of *Parasutterella* was significantly higher in the AX group and the OBG group compared to CTRL (uncorrected *p* = 0.005 and *p* = 0.049, respectively). These significant differences were still significant after correction for multiple testing. At phylum level, the Bacteroidetes:Firmicutes ratio was not significantly different between the groups.

### 3.6. Short-Chain Fatty Acids and Faecal pH

No significant changes in any of the faecal SCFA levels were observed throughout the intervention period between the OBG, AX and CTRL groups (Table S3; all *p* ≥ 0.129), while a significantly lower increase in faecal pH was measured in the AX group compared to CTRL (uncorrected *p* = 0.005) that was still significant after correction for multiple testing.

### 3.7. Dietary Intake

During the study period, no significant changes in dietary intake were observed between the NPS intervention groups over time (Table S4; all *p* ≥ 0.386). From two weeks prior to starting the intervention, subjects were asked to reduce their dietary fibre intake. Based on the dietary intake data, the fibre intake within the complete study population was indeed significantly decreased during the intervention compared to the period before wash-out (18.26 [14.16; 22.38] vs. 20.60 [16.56; 20.09]; *p* < 0.001).

### 3.8. Gastrointestinal Symptom Scores, Stool Frequency and Stool Consistency

The gastrointestinal symptom scores and stool frequency did not significantly change over time between groups, indicating that the NPS products were well tolerated (Table S5). Compared to the CTRL group, Bristol stool consistency scores decreased after supplementation with YBG, SBG, and AX (all uncorrected and corrected *p* ≤ 0.025) and tended to decrease in the OBG group (uncorrected *p* = 0.087), indicating softer stools.

### 3.9. Laboratory Safety Parameters and Adverse Events

During the complete study period, no statistically significant changes in laboratory safety parameters occurred over time between the intervention groups (Table S6). Furthermore, NPS supplementation did not significantly affect the total number of adverse events, the total number of RTI, the total number of vaccination side effects or the total number of constipation-related, diarrhoea-related, or other adverse events (data not shown).

## 4. Discussion

The trend in increase of HI antibody titres and seroprotection rate against the influenza A H1N1 strain in response to vaccination in subjects consuming AX compared to CTRL might suggest an adjuvant effect of AX on the immune response to vaccination against this influenza strain in seniors. An effect of orally administered AX on the antibody-mediated immune response has been reported previously in chickens [31]. Although the underlying mechanism has not been clarified yet, several immunomodulatory activities of AX could explain this effect. Sun et al. [51] demonstrated that oral treatment with carrot pomace-derived polysaccharides could enhance vaccine-specific antibody titres in immunosuppressed mice and hypothesised that food-derived polysaccharides could enhance the antigen presentation capacity of innate immune cells (e.g., macrophages and dendritic cells) and increase dendritic cell maturation. Indeed, AX has been reported to activate dendritic cell maturation [16] and stimulate the expression of the C-type lectin receptor DEC-205, which facilitates antigen presentation by dendritic cells in vitro [52]. Moreover, AX has been found to enhance macrophage phagocytic activity in vitro [15,53]. Tang et al. [54] and Govers et al. [55] studied the same NPS as utilised in the present trial through in vitro models. These studies showed that AX was the most potent NPS in supporting macrophage differentiation into a specific subtype [54]. Among the NPS tested, AX also showed the strongest induction of transcription and secretion of a unique set of cytokines and chemokines and an increase in monocyte recruitment capacity of macrophages, suggesting enhanced immune cell vigilance [55]. Given that vaccine efficacy is dependent on the host’s ability to rapidly recruit competent innate immune cells [51], this could explain why AX may effectively boost vaccine-mediated antibody responses in an older population.

The observed effects of AX on HI antibody titres seemed to be “strain-dependent”: the enhancement was most pronounced for the influenza A H1N1 strain. A recent meta-analysis by Yeh et al. [56] supports these results by showing that prebiotic supplementation (mainly galacto- and fructo-oligosaccharides) for eight or more weeks could significantly increase HI antibody titres against the H1N1 strain after influenza vaccination, while a similar but non-significant overall effect was observed for HI antibody titres against the H3N2 and B strains. Despite the similarity with the NPS tested herein, and AX in particular, it is difficult to explain this strain-specific difference in vaccine-enhancing effects. However, it is known that prior vaccinations and natural infections with closely related influenza strains could affect pre-vaccination antibody titres and antibody response to vaccination [57]. We observed higher pre-vaccination antibody titres for the H1N1 strain compared to the other influenza strains, which is most likely the result of cross-reactive antibodies from prior vaccination of the subjects during previous influenza seasons. Indeed, a recent study investigating the impact of repeated annual vaccination against the same influenza A (H1N1)pdm09 strain also reported elevated pre-vaccination antibody titres and augmentation of HI antibody responses three weeks after repeated vaccination [57]. In contrast, repeated annual vaccination could blunt the HI antibody response to influenza vaccine strains that undergo frequent antigenic changes, particularly H3N2 [58]. While the H1N1 strain in the 2012–2013 influenza vaccine administered in this study was antigenically homogeneous to the H1N1 strain used in the 2010–2011 and 2011–2012 influenza vaccines, the H3N2 and influenza B strains were antigenically different [40,59,60], explaining the lower antibody cross-reactivity and pre-vaccination antibody titres for these latter strains compared to H1N1 in the current study. Although the micro-neutralization assay is considered to be more sensitive than the HI assay for detection of antibody titres, cross-reactive antibodies can limit the interpretation of this assay similarly [61].

In the HI as well as the micro-neutralization assay, a lower fold increase in H1N1 antibody response to vaccination was observed in subjects consuming SBG compared to CTRL, which might be explained by the high pre-vaccination antibodies against H1N1 in the SBG group at V1. Hence, the vaccine-mediated antibody response might have appeared less pronounced in this group. The variable pre-vaccination antibody titres across intervention groups most probably is due to the small sample size of this study, which exposes the results to the risk of random effects. As reported by multiple animal studies investigating the adjuvant effects of SBG in a vaccination model, SBG could significantly increase vaccine-specific antibody responses [28,29]. Moreover, a human crossover trial showed that ingestion of SBG at a daily dosage of 2.5 mg for six weeks could increase the number of circulating B-cells in an older population aged 50 years and older [62] and recent in vitro analyses based on macrophages indicated that SBG shows similar, albeit less potent, immunomodulatory activities to AX [15,55]. In line with these findings, we found a clear but non-significant trend towards less common cold episodes during SBG consumption in the current study, suggesting a protective effect of SBG against viral infections. Due to the conflicting results, it remains to be established whether SBG indeed has immunomodulatory effects in humans and additional studies with higher dosages and, particularly, larger sample sizes are required. 

On the contrary, the observed beneficial effects of AX on the immune response are further supported by exploratory data of our study indicating a lower incidence of common colds during the five-week AX consumption period, although statistical significance was not achieved with the pilot-powered trial. Similarly, Maeda et al. [63] reported a reduced duration and severity of common cold symptoms in elderly participants during a six-week treatment with AX derivatives, accompanied by an increase in natural killer (NK) cell activity. Enhancing NK cell activity might increase the resistance to viral infections in the elderly population, since NK cells constitute the first line of defence against virally infected cells and an age-dependent decrease in NK cell immunity has been reported [64]. Recently, AX consumption (500 mg/day) for one month has been shown to induce a significant increase in NK cell activity in elderly subjects [65]. Although NK cell activity was not studied in our pilot trial, we observed an effect of AX on IFN-γ, a key cytokine produced by NK cells [66]. The results of cytokine analyses during the intervention indicated that AX, as well as other NPS such as OBG, YBG, and EPS could counteract a decrease in serum IFN-γ levels, which was observed in the CTRL group. These results are in agreement with previous studies reporting that supplementation with an AX-rich substance for eight weeks and YBG for 10 days could increase serum IFN-γ levels in healthy subjects [18,67], although others did not show this effect for YBG [68]. 

Emerging evidence reveals that probiotics, prebiotics, and the intestinal microbiota can significantly influence the immune response to vaccination and the incidence of infectious diseases in the host, particularly respiratory virus infections [4,12,69,70]. In our study, we showed that subjects consuming AX for five weeks had a higher relative abundance of *Bifidobacterium* compared to subjects in the CTRL group. Therefore, AX appeared to have a bifidogenic effect, which is supported by previous studies reporting a prebiotic effect of AX on *Bifidobacterium* species [11,71,72]. Several *Bifidobacterium* species (e.g., *B. longum*, *B. breve* and *B. bifidum*) have been identified to reduce the incidence of influenza infections and reduce the severity and duration of common cold episodes in humans [70,73,74,75]. Thus, we speculate that the lower incidence of common colds during AX consumption in our study might be attributed, at least in part, to a relative increase in intestinal *Bifidobacterium* species. The higher faecal *Bifidobacterium* abundance after AX consumption could be expected to be associated with a change in SCFA levels and faecal pH, as found in previous piglet trials [71,76]. However, despite a significantly lower increase in faecal pH in the AX group compared to the CTRL, which is potentially related to the subjects’ low dietary fibre consumption and abstinence from pro- and prebiotic foods during intervention, no changes in SCFA levels were observed in our study. Whereas the role of *Bifidobacterium* species is well established in the context of mediating immunomodulatory effects, the role of *Parasutterella* is still to be clarified. Some recent studies, however, indicated that *Parasutterella* is also associated with immunity [77,78,79]. Thus, since AX at a daily dosage of 10 g had an impact on intestinal microbiota and showed some immunomodulatory effects, the involvement of microbiota and a prebiotic role was likely contributory to or mediating its immunomodulatory actions. The wheat endosperm AX used in this clinical trial was characterised by less complex side chains compared to AX isolated from other parts of the grain [30]. Although it is not clear yet whether structural characteristics of AX influence their prebiotic properties, one could speculate that AX with less complex side chains might be more efficiently metabolized by beneficial intestinal bacteria. However, the impact of the specific structural features of AX on its prebiotic effects warrants further research.

No adverse effects or gastrointestinal side effects of NPS consumption were reported by subjects in this study, indicating that the NPS products were well-tolerated and oral NPS supplementation is a safe and feasible intervention. Moreover, consumption of most of the NPS could soften stools, which is generally considered a beneficial physiological effect associated with the water binding capacity of NPS [6,80]. However, for YBG and SBG, the dosage of 500 mg may be considered as too low to exert a relevant water binding effect. Based on the improvement of stool consistency, NPS could, besides immune support, also provide gut health and bowel support with no adverse side effects.

Some potential limitations of this study should be mentioned. First of all, this study was designed as a pilot study, as no data on the primary outcome (i.e., HI titres after influenza vaccination) from previous studies with these NPS were available to estimate an adequate sample size. Due to the inclusion of multiple intervention groups (i.e., multiple comparisons) and relatively small group sizes, the study was underpowered, and the results must be interpreted with caution. Instead, positive trends and close-to-significant findings of this study should be regarded indicative of effects that should be verified in a confirmatory, large-scale randomised trial, for which it was calculated that over 110 subjects per intervention arm would be required to detect stimulatory effects of AX on vaccine-mediated increases in HI seroprotection rate against influenza A H1N1. Secondly, administered dosages of NPS differed between the intervention groups, as the chosen dosages used in this trial were based on recommended dosages by the respective producers of the NPS. 

Therefore, we cannot directly compare the immunomodulatory effects of the different NPS, as the observed outcome differences may result from dosage inequalities. In case of those NPS, which were administered in dosages below 10 g daily, prebiotic effects could not be expected [81] and merely direct interactions with host cells might have been assumed as the basis for immunomodulatory effects. Further studies with higher and/or equal NPS dosages should be conducted to compare immunomodulatory effects and enable comparison of effect sizes between NPS. Thirdly, we observed a large interindividual variation for all outcomes in this study, which may have clouded possible small beneficial effects of the NPS interventions. This interindividual variation could be a consequence of the older population and the variation in previous history of influenza vaccinations or natural infections. As antigenic similarity between previous influenza vaccine strains or circulating strains and current influenza vaccine strains is known to affect antibody titres and vaccine efficacy, adjusting for these factors might be required to evaluate the effectiveness of NPS to enhance the immune response. The existence of this memory factor raises the question of whether the best strategy to investigate the adjuvant effects of NPS in the elderly would be to use vaccines to which the study subjects and their immune systems are naïve. Finally, although the primary focus of this trial was to target immunosenescence in the older population, the immunocompetence within the population studied might differ largely as a consequence of the relatively wide age range of included subjects. This may have played a role in the interindividual variation that was observed, as immunocompetent subjects may have had less pronounced responses to the interventions than subjects with an impaired immune function.

Although the explorative data of this pilot study were subject to a large interindividual variation and should be interpreted with caution, the results suggest that AX supplementation at a daily dosage of 10 g can be feasible, tolerable, and safe as an oral adjuvant to support vaccine efficacy and protection against viral infections in elderly individuals. However, additional large-scale clinical trials should be conducted in the future to confirm this.

## Figures and Tables

**Table 1:** Baseline characteristics of the study population.

	Total (*n* = 239)	YBG (*n* = 40)	SBG (*n* = 40)	OBG (*n* = 40)	AX (*n* = 40)	EPS (*n* = 39)	CTRL (*n* = 40)	*p*-Value
Female/male (*n*)	118/121	17/23	19/21	23/17	22/18	23/16	14/26	0.212
Age (years)	67.9 [63.1; 71.4]	68.2 [62.1; 71.2]	67.3 [60.2; 70.0]	68.0 [63.0; 71.3]	67.5 [63.7; 71.3]	66.2 [62.2; 72.3]	69.5 [65.0; 73.3]	0.271
BMI (kg/m^2^)	27.0 ± 3.6	27.2 ± 3.9	27.0 ± 3.1	26.9 ± 3.5	26.7 ± 4.0	27.3 ± 3.7	27.3 ± 3.5	0.969

Values are presented as numbers, medians [Q1; Q3], or mean ± SD. Differences in sex between intervention groups were tested with a Chi-square test. Differences in age were tested with a Kruskal–Wallis test and differences in BMI were tested with one-way ANOVA. BMI: body mass index, YBG: yeast β-glucan, SBG: shiitake β-glucan, OBG: oat β-glucan, AX: arabinoxylan, EPS: exopolysaccharide, CTRL: control.

**Table 2:** Haemagglutination inhibition (HI) titres at baseline (V1) and three weeks after vaccination (V4) in the different NPS intervention groups.

		YBG	SBG	OBG	AX	EPS	CTRL	*p*
GMT (Log_2_(HI Titre/10))								
H1N1	V1	1.00 [0.00; 3.00]	1.75 [0.00; 3.00]	0.50 [0.00; 2.50]	0.88 [−0.44; 2.00]	1.50 [0.00; 2.50]	1.38 [0.13; 2.94]	0.557
	V4	2.88 [1.00; 4.00]	2.50 [1.00; 3.50]	2.50 [0.50;3.50]	2.50 [1.88; 3.63]	2.00 [1.00; 3.00]	3.00 [1.50; 4.00]	0.548
	MLFI	1.00 [0.00; 1.50]	0.75 [0.00; 1.50] *	1.00 [0.25; 2.25]	2.00 [1.00; 3.00] ^#^	1.00 [0.50; 2.00]	1.25 [0.50; 2.00]	0.010
H3N2	V1	0.00 [−1.00; 0.69]	0.00 [−1.00; 0.50]	0.00 [−1.00; 0.50]	−0.38 [−1.00; 0.50]	−0.50 [−1.00; 0.00]	−0.25 [−1.00; 0.44]	0.266
	V4	1.00 [−1.00; 2.00]	1.00 [0.00; 1.50]	1.00 [0.25; 1.50]	1.00 [0.00; 2.00]	0.50 [0.00; 1.25]	1.00 [0.50; 1.75]	0.382
	MLFI	0.50 [0.00; 1.50]	0.75 [0.00; 1.25]	1.00 [0.50; 2.00]	1.00 [0.38; 1.75]	1.00 [0.00; 1.75]	1.00 [0.50; 2.00]	0.198
Influenza B	V1	−0.50 [−1.00; 2.00]	−0.50 [−1.00; 0.50]	0.00 [−1.00; 0.44]	−0.25 [−1.00; 1.00]	0.00 [−1.00; 1.00]	0.13 [−1.00; 1.88]	0.741
	V4	1.00 [1.19; 2.06]	1.00 [0.00; 2.00]	1.00 [0.00; 1.50]	1.00 [0.00; 2.13]	1.00 [−0.25; 2.00]	1.00 [0.00; 2.00]	0.786
	MLFI	1.00 [0.00; 2.06]	1.00 [0.00; 2.00]	0.75 [0.00; 1.75]	1.00 [0.00; 2.00]	0.75 [0.00; 2.00]	1.00 [0.00; 1.50]	0.683

Values are presented as medians [Q1; Q3]. *p* represents the *p*-values for the differences at each visit (V1 and V4) and over time (V1 to V4) between the six intervention groups tested with a Kruskal–Wallis test. * represents *p* < 0.05 for the pairwise comparisons for differences between V1 and V4 compared to CTRL, tested by uncorrected Mann–Whitney U test. ^#^ represents a *p* < 0.1 for the pairwise comparisons for differences between V1 and V4 compared to CTRL, tested by uncorrected Mann–Whitney U test. NPS: non-digestible polysaccharide, GMT: geometric mean titre, MLFI: mean log_2_ fold increase between V1 and V4, YBG: yeast β-glucan, SBG: shiitake β-glucan, OBG: oat β-glucan, AX: arabinoxylan, EPS: exopolysaccharide, CTRL: control.

**Table 3:** Changes in seroprotection and seroconversion rate in the haemagglutination inhibition (HI) assay between baseline (V1) and three weeks after vaccination (V4) in the different NPS intervention groups.

		YBG	SBG	OBG	AX	EPS	CTRL	*p*
Seroprotection rate (%)								
H1N1	V1	42.5	50.0	27.5	27.5	38.5	42.5	0.230
	V4	60.5	59.0	59.0	75.7	61.5	66.7	0.633
	V4-V1	18.4	10.3	30.8	48.7 ^#^	23.1	25.6	0.005
H3N2	V1	5.0	7.5	2.5	7.5	0.0	2.5	0.476
	V4	26.3	17.9	19.7	32.4	15.4	20.5	0.468
	V4-V1	21.0	12.8	15.4	24.3	15.4	17.9	0.802
Influenza B	V1	15.0	7.5	7.5	10.0	10.3	25.0	0.148
	V4	36.8	28.2	17.9	37.8	25.6	35.9	0.343
	V4-V1	26.3	23.1	10.3	27.0	15.4	10.3	0.180
Seroconversion rate (%)								
H1N1	V1 to V4	15.8	17.9	28.2	43.2	17.9	23.1	0.056
H3N2	V1 to V4	13.2	7.7	10.3	10.8	10.3	17.9	0.799
Influenza B	V1 to V4	21.1	17.9	7.7	21.6	17.9	15.4	0.618

Values are presented as proportions (%). *p* represents the *p*-values for the differences at each visit (V1 and V4) and over time (V1 to V4) between the six intervention groups tested with a Chi-square test. ^#^ represents a *p* < 0.1 for the pairwise comparisons for differences between V1 and V4 compared to CTRL, tested by uncorrected Fisher exact test. NPS: non-digestible polysaccharide, YBG: yeast β-glucan, SBG: shiitake β-glucan, OBG: oat β-glucan, AX: arabinoxylan, EPS: exopolysaccharide, CTRL: control.

**Table 4:** Micro-neutralization titres at baseline (V1) and three weeks after vaccination (V4) in the different NPS intervention groups.

		YBG	SBG	OBG	AX	EPS	CTRL	*p*
GMT (Log_2_(Titre/10))								
H1N1	V1	0.49 [−0.46; 3.88]	2.51 [−0.51; 4.50]	0.75 [−0.51; 2.18]	0.49 [−0.51; 2.38]	1.00 [−0.51; 2.51]	0.49 [−0.51; 2.00]	0.408
	V4	4.50 [2.88; 6.00]	4.50 [3.00; 6.13]	4.50 [2.74; 6.00]	5.50 [3.75; 6.00]	4.50 [2.00; 5.50]	5.00 [3.00; 6.50]	0.437
	MLFI	2.00 [0.93; 4.26] ^#^	1.50 [0.51; 3.63] *	3.51 [1.74; 5.01]	3.77 [2.50; 5.51]	2.09 [1.00; 4.50]	3.50 [1.50; 5.51]	0.013
H3N2	V1	1.25 [0.49; 3.88]	1.49 [0.00; 2.51]	0.49 [−0.51; 1.49]	0.49 [−0.51; 2.00]	0.49 [−0.51; 1.49]	0.49 [0.00; 2.00]	0.031
	V4	4.25 [1.87; 5.63]	4.50 [2.51; 5.50]	4.00 [1.49; 6.00]	4.50 [2.76; 6.00]	3.50 [2.51; 5.00]	4.00 [3.00; 5.50]	0.586
	MLFI	1.88 [0.51; 3.50]	2.02 [0.88; 4.01]	3.01 [1.49; 5.50]	3.00 [1.00; 4.76]	3.03 [1.51; 4.50]	3.03 [1.51; 5.02]	0.112
Influenza B	V1	1.00 [0.13; 1.50]	1.00 [0.50; 2.00]	0.50 [0.00; 1.50]	1.00 [0.50; 2.00]	0.50 [0.00; 2.00]	1.00 [0.50; 2.00]	0.486
	V4	3.00 [2.50; 4.50]	3.25 [2.00; 4.55]	3.00 [1.50; 4.00]	3.50 [1.90; 5.15]	3.50 [2.00; 5.30]	3.80 [2.00; 4.50]	0.609
	MLFI	2.03 [0.95; 3.51]	1.52 [0.87; 3.01]	1.81 [1.00; 3.01]	2.03 [1.49; 3.37]	2.26 [1.10; 3.52]	2.00 [0.51; 3.01]	0.745

Values are presented as medians [Q1; Q3]. *p* represents the *p*-values for the differences at each visit (V1 and V4) and over time (V1 to V4) between the six intervention groups tested with a Kruskal–Wallis test. * represents *p* < 0.05 for the pairwise comparisons for differences between V1 and V4 compared to CTRL, tested by uncorrected Mann–Whitney U test. ^#^ represents a *p* < 0.1 for the pairwise comparisons for differences between V1 and V4 compared to CTRL, tested by uncorrected Mann–Whitney U test. NPS: non-digestible polysaccharide, GMT: geometric mean titre, MLFI: mean log_2_ fold increase, YBG: yeast β-glucan, SBG: shiitake β-glucan, OBG: oat β-glucan, AX: arabinoxylan, EPS: exopolysaccharide, CTRL: control.

**Table 5:** Changes in seroprotection and seroconversion rate in the micro-neutralization assay between baseline (V1) and three weeks after vaccination (V4) in the different NPS intervention groups.

		YBG	SBG	OBG	AX	EPS	CTRL	*p*
Seroprotection rate (%)								
H1N1	V1	40.0	53.8	25.0	35.0	33.3	30.0	0.130
	V4	86.8	84.2	84.6	89.2	79.5	89.7	0.813
	V4-V1	44.7	30.8 *	59.0	59.5	46.2	61.5	0.050
H3N2	V1	37.5	38.5	15.0	32.5	20.5	27.5	0.126
	V4	76.3	86.8	74.4	81.1	84.6	97.4	0.082
	V4-V1	39.5	48.7	59.0	48.6	64.1	71.8	0.053
Influenza B	V1	22.5	30.8	20.0	32.5	30.8	30.0	0.753
	V4	84.2	78.9	74.4	75.7	79.5	76.9	0.927
	V4-V1	60.5	50.0	53.8	43.2	48.7	46.1	0.726
Seroconversion rate (%)								
H1N1	V1 to V4	52.6	39.5 ^#^	69.2	75.7	51.3	61.5	0.019
H3N2	V1 to V4	42.1	52.6	64.1	56.8	69.2	71.8	0.076
Influenza B	V1 to V4	60.5	47.4	48.7	56.8	53.8	51.3	0.863

Values are presented as proportions (%). *p* represents the *p*-values for the differences at each visit (V1 and V4) and over time (V1 to V4) between the six intervention groups tested with a Chi-square test. * represents *p* < 0.05 for the pairwise comparisons for differences between V1 and V4 compared to CTRL, tested by uncorrected Fisher exact test. ^#^ represents a *p* < 0.1 for the pairwise comparisons for differences between V1 and V4 compared to CTRL, tested by uncorrected Fisher exact test. NPS: non-digestible polysaccharide, YBG: yeast β-glucan, SBG: shiitake β-glucan, OBG: oat β-glucan, AX: arabinoxylan, EPS: exopolysaccharide, CTRL: control.

**Table 6:** Number of subjects with common cold episodes during the intervention period (V1–V4).

*n*	YBG	SBG	OBG	AX	EPS	CTRL	*p* *Chi-Square Test*	*p* *Fisher Exact Test* AX vs. CTRL
Cold acc. to Predy et al. [43]	3	1	3	1	5	5	0.317	0.113
Cold acc. to Jackson et al. [44]	7	3	5	1	7	8	0.141	0.029

Values represent the number of subjects with colds diagnosed based on Predy criteria or Jackson criteria, respectively. *p Chi-square test* represents the *p*-values for the comparison between the six intervention groups tested with a Chi-square test. Pairwise comparisons were performed using Fisher exact test to calculate *p*-values comparing each intervention group with CTRL. With the exception of AX, all pairwise comparisons were not significant (*p* > 0.05). *p Fisher exact test* represents *p*-values for the pairwise comparisons comparing AX and CTRL, tested by uncorrected Fisher exact test. YBG: yeast β-glucan, SBG: shiitake β-glucan, OBG: oat β-glucan, AX: arabinoxylan, EPS: exopolysaccharide, CTRL: control.

## Data Availability

Pseudonymised data are available on request from the corresponding author.

## Supplementary Materials

The following are available online at https://www.mdpi.com/article/10.3390/nu13082683/s1: Methods S1: Eligibility criteria; Methods S2: preparation method shiitake β-glucan; Methods S3: preparation method exopolysaccharide; Table S1: Chemical characterisation of the NPS; Table S2: Cytokine production in whole blood after incubation with medium, Concanavalin A, or lipopolysaccharide at baseline, before vaccination and one week after vaccination in the different NPS intervention groups; Table S3: Faecal short-chain fatty acid levels and faecal pH at baseline and after five weeks of NPS consumption; Table S4: Dietary intake at screening and after five weeks of NPS consumption; Table S5: GI symptom scores, stool frequency, and stool consistency at baseline and after five weeks of NPS consumption; Table S6: Laboratory safety parameters at screening and after five weeks of NPS consumption.
