# Supplementary material for: Adjuvant Effect of Orally Applied Preparations Containing Non-Digestible Polysaccharides on Influenza Vaccination in Healthy Seniors: A Double-Blind, Randomised, Controlled Pilot Trial
Source: Nutrients. 2021 Aug 2;13(8):2683. doi: 10.3390/nu13082683 (PMC8400163; doi:10.3390/nu13082683)
Supplement: Supplementary file 1 [file nutrients-13-02683-s001.zip › nutrients-1309852-Supplementary.pdf]

Supplementary material

Methods S1. Eligibility criteria

Exclusion criteria were concomitant enrolment in another clinical study within the last four weeks, previous vaccination against influenza during 2012-2013 with either the influenza vaccine used in the present study or another influenza vaccine, vaccination against influenza within the previous 10 months, suffering from influenza or influenza-like illness within the previous 10 months, other vaccinations during and within two months before the study, symptoms of common infectious diseases of the airways or other organ systems e.g. gastrointestinal tract in the last four weeks before inclusion, known congenital, acquired or iatrogenic immunodeficiency (e.g., HIV, chemotherapy, immunosuppression), active autoimmune diseases, allergy or hypersensitivity to any component of the vaccine (e.g., chicken egg protein, certain antibiotics), to any component of the test product (e.g., yeast, gluten, shiitake mushrooms) or to latex, known coeliac disease (gluten enteropathy), bowel movement less than three times per week, fulfilment of two or more Rome III criteria for constipation or taking laxatives on a regular basis, severe chronic disease (cancer, inflammatory bowel disease, malabsorption, maldigestion (including lactose intolerance), malnutrition, chronic inflammatory diseases, renal, hepatic or cardiac diseases, COPD, respiratory insufficiency), chronic abdominal pain, malformation of fingers, systemic treatment or topical treatment likely to interfere with evaluation of the study parameters, such as antibiotics, intestinal or respiratory antiseptics, anti-rheumatics, antiphlogistics (except aspirin or equivalent products preventing from aggregation of platelets or blood clotting) and steroids prescribed in chronic inflammatory diseases, other treatments likely to interfere with study outcome (e.g., laxatives, body weight management and/or medication), treatments with calcium antagonists and nitrates and alpha blockers, diabetes mellitus on drug therapy, severe neurological, cognitive or psychiatric diseases, surgery or intervention requiring general anaesthesia within two months before the study, eating disorders or special diets (e.g., anorexia, bulimia, vegetarian, vegan), alcohol and drug abuse, pregnancy or lactation, legal incapacity, anaemia (defined as haemoglobin < 12

g/dL), liver damage (defined as ALAT or ASAT > two-fold increased) or kidney damage (serum creatinine > 1.2 mg/dL).

Methods S2. Preparation method shiitake β-glucan

The β-glucan of shiitake was isolated from commercial shiitake produced by Paulissen-Evert, a commercial shiitake grower in Swolgen, the Netherlands. No pesticides were used during growing of the mushrooms. In short: sawdust substrate bags (2.8 kg) were inoculated with 30 ml spawn. This spawn was prepared by inoculating pure cultures on sterilized sorghum grain. The bags were incubated using the climate parameters: 20°C, 1500ppm CO_2 and 90% RH. After six weeks of vegetative growth, the plastic was removed from the blocks and the blocks were placed in a production room. The conditions for fruiting body production were 16°C, 1000ppm CO_2, 90% RH and 12 hours light a day. Fruiting body production started after six weeks of vegetative growth. Fruiting bodies were harvested at mature size and transported by special courier (which was used for all shipments of the grower) only transporting the mushrooms and no other cargo and delivered directly to The RijnIJssel (Wageningen, The Netherlands). The isolation of the β-glucans from shiitake was performed at the kitchen of the regional training centre and education school The RijnIJssel by Wageningen Food and Biobased Research (WFBR) employees. Kitchen rules were according to HACCP rules. All persons (all employed at WFBR) used the standard precautions for food preparations like washing hands before entering the facility, wearing disposable coats, hear nets, no jewels, use of blue plaster if needed etc. The fresh shiitake mushrooms were homogenized with standard kitchen apparatus and put in 100 litre soup kettle and boiled in standard tap water. The ratio was 33 kg fresh shiitake homogenized in 50 litre of tap water. The homogenized mushrooms were boiled at 100˚C for eight hours and then cooled down to room temperature. The homogenate was then filtered over cheesecloth (bought from cheese factory for cheese filtering purposes) by centrifugation in a new centrifuge. To 50 litre of filtered and almost clear extract, 50 litre 96% food grade ethanol was added and thoroughly stirred in a 100 litre soup kettle. The total extract was placed overnight in the locked kitchen at 18 °C. After an overnight incubation the upper phase of floating and clustered β-glucan fibres was taken from the surface of the extract and put into a

colander. After several hours of leakage of liquid content, the fibre fraction was frozen at -80 °C in closed boxes and transported to WFBR. Subsequently, the frozen fibre fraction was freeze dried in a thoroughly cleaned freeze drier at the food lab of WFBR. The dried fibre was grinded using a standard kitchen machine into a fine powder. No harmful pathogens have been found in the product and also aerobic mesophilic plate count (ISO 4833) only resulted in very low numbers of cfu/g material, indicating that the product was safe for use.

Methods S3. Preparation method exopolysaccharide

The exopolysaccharide (EPS) was isolated from *Lactobacillus mucosae* DPC 6426, a lactic acid bacterium from mammalian gut origin previously isolated at Teagasc Moorepark Food Research Centre (Fermoy, Ireland). This strain exhibited a very ropy phenotype, indicating EPS production when grown on de Man, Rogosa and Sharpe (MRS) agar containing 5% (w/v) glucose. In short: *Lactobacillus mucosae* DPC 6426 was activated and propagated in MRS medium with the addition of 5% (w/v) sucrose at 37°C anaerobically. pH controlled (pH 6) fermentation was undertaken in a newly developed food-grade medium consisting of 10% (w/v) whey permeate, 5% (w/v) sucrose and 2% (w/v) whey protein hydrolysate at 30°C aerobically for 18 hours. Following fermentation, *Lactobacillus mucosae* DPC 6426 was inactivated by adding 8% (v/v) of 20% (w/v) trichloroacetic acid (TCA) solution and to precipitate protein. To remove precipitated proteins and bacterial cells, solution was separated 1x g and supernatant containing EPS was adjusted to pH 6.8 by adding 35 % (w/v) NaOH. To purify and concentrate EPS, the supernatant was ultrafiltrated/diafiltrated using a 5kD membrane following evaporation of excess water at 60°C. Resulting retentate was spray-dried with an inlet temperature of ~175°C and outlet temperature of ~90°C. The spray-dried powder was silky with no distinctive odour and a light yellowish colour. Approximately 4.5 kg EPS powder was obtained from 800 litre fermentation medium with a purity of 80% EPS. The EPS powder was cleared microbiologically using 25kdose irradiation.

Table S1. Chemical characterisation of the NPS (data partly adapted from [36]).

	YBG	SBG	OBG	AX	EPS
Mono sugar composition					
Rha (%)	0.5	0.3	0.4	0.2	0.0
Fuc (%)	0.0	0.9	0.0	0.0	0.0
Ara (%)	0.0	1.8	5.6	24.5	0.0
Xyl (%)	0.0	0.4	8.6	29.5	0.0
Man (%)	0.0	4.6	0.9	1.2	0.3
Gal (%)	0.0	4.0	0.8	11.8	17.7
Glc (%)	93.1	59.0	45.7	9.2	31.6
NAcGlcN (%)					9.6
UA (%)	1.0	1.6	1.7	1.0	0.1
Total sugar (%)	94.6	72.7	63.7	77.4	49.8
Main basal structure	β-1,3-Glc backbone with short (4-6) side chains via β-1,6-linkages	β-1,3-Glc backbone and β-1,6-linkages Glc ramifications together with some starch β-1,4- and 1,4,6-Glc linkages	β-1,3/1,4-linked β-glucan	Arabinoxylans and additional arabinogalactan peptides together with some β-1,3/1,4-linked β-glucans and β-1,4-mannans	XX
MW (kDa)	~17	~21000	~600	~95	XX
Protein (%)	0.5	16.2	23.8	7.7	n.a.
Starch (%)	0.8	8.6	8.0	0.6	n.a.
Solubility	soluble	partly soluble	soluble after preheating	soluble	partly soluble
Total prep	95.9	97.5	95.5	85.7	49.8

Values are presented as % dry weight of NPS. NPS: non-digestible polysaccharide, Rha: rhamnose, Fuc: fucose, Ara: arabinose, Xyl: xylose, Man: mannose, Gal: galactose, Glc: glucose, NAcGlcN: N-acetyl glucosamine, UA: uronic acid, Total sugar: sum of the mono sugars, MW: molecular weight, kDa: kilodalton, n.a.: not applicable, YBG: yeast β-glucan, SBG: shiitake β-glucan, OBG: oat β-glucan, AX: arabinoxylan, EPS: exopolysaccharide.

Table S2. Cytokine production in whole blood after incubation with medium, Concanavalin A (ConA) or lipopolysaccharide (LPS) at baseline (V1), before vaccination (V2) and one week after vaccination (V3) in the different NPS intervention groups

		YBG	SBG	OBG	AX	EPS	CTRL	*P*
**Medium**								
**IFN-y (pg/ml)**	**V1**	3.37 [2.49; 10.59]	3.37 [2.49; 10.59]	3.37 [1.26; 10.59]	3.61 [0.67; 11.30]	4.29 [2.15; 8.03]	5.40 [3.05; 10.59]	
	**V2**	8.70 [1.94; 13.34]	5.51 [3.05; 10.71]	3.05 [1.94; 10.79]	6.73 [2.95; 17.02]	5.70 [3.02; 14.86]	4.21 [2.78; 10.99	0.275
	**V3**	3.31 [2.46; 13.20]*	2.90 [0.63; 8.70]	4.91 [2.32; 20.11]*	3.31 [2.32; 22.31]*	6.35 [2.43; 13.62]*	3.31 [1.22; 11.52]	0.013
**TNF-α (pg/ml)**	**V1**	0.75 [0.54; 1.44]	0.67 [0.54; 0.78]	0.70 [0.63; 2.09]	0.85 [0.60; 1.17]	0.79 [0.60; 1.59]	0.62 [0.38; 0.85]	
	**V2**	1.67 [0.78; 3.15]	1.02 [0.69; 3.15]	2.15 [0.35; 3.15]	2.15 [0.82; 3.15]	1.64 [0.69; 2.49]	1.63 [0.69; 3.15]	0.364
	**V3**	0.78 [0.62; 1.58]	0.78 [0.56; 2.92]	0.78 [0.68; 2.87]	0.85 [0.58; 2.92]	0.96 [0.60; 2.32]	0.77 [0.56; 1.69]	0.408
**IL-1β (pg/ml)**	**V1**	0.15 [0.09; 0.27]	0.17 [0.12; 0.23]	0.19 [0.13; 0.25]	0.16 [0.10; 0.27]	0.15 [0.10; 0.25]	0.16 [0.11; 0.23]	
	**V2**	0.14 [0.11; 0.31]	0.19 [0.14; 0.28]	0.18 [0.14; 0.39]	0.23 [0.13; 0.63]	0.18 [0.10; 0.29]	0.16 [0.11; 0.29]	0.654
	**V3**	0.13 [0.08; 0.24]	0.19 [0.06; 0.35]	0.16 [0.08; 0.30]	0.13 [0.06; 0.58]	0.14 [0.09; 0.23]	0.15 [0.09; 0.22]	0.802
**IL-2 (pg/ml)**	**V1**	0.10 [0.08; 0.94]	0.10 [0.08; 0.99]	0.10 [0.08; 0.32]	0.11 [0.10; 0.39]	0.10 [0.08; 0.34]	0.11 [0.09; 0.39]	
	**V2**	0.33 [0.11; 0.34]	0.31 [0.09; 0.33]	0.31 [0.10; 0.34]	0.33 [0.09; 0.36]	0.33 [0.09; 0.34]	0.33 [0.10; 0.34]	0.765
	**V3**	0.09 [0.08; 0.31]	0.09 [0.08; 0.34]	0.09 [0.08; 0.31]	0.09 [0.08; 0.34]	0.09 [0.07; 0.31]	0.10 [0.08; 0.31]	0.583
**IL-12 (pg/ml)**	**V1**	0.26 [0.25; 1.03]	0.26 [0.25; 1.01]	0.26 [0.21; 1.10]	0.26 [0.25; 1.10]	0.26 [0.21; 0.98]	0.30 [0.25; 1.12]	
	**V2**	0.30 [0.25; 1.02]	0.29 [0.24; 1.01]	0.29 [0.24; 1.02]	1.01 [0.25; 1.28]	0.29 [0.25; 0.78]	0.48 [0.27; 1.04]	0.728
	**V3**	0.68 [0.23; 1.04]	1.02 [0.30; 1.04]	0.96 [0.23; 1.04]	0.68 [0.23; 1.06]	0.30 [0.23; 1.03]	0.68 [0.26; 1.05]	0.636
**IL-10 (pg/ml)**	**V1**	0.43 [0.27; 0.58]	0.40 [0.23; 0.78]	0.55 [0.32; 0.98]	0.48 [0.32; 0.90]	0.42 [0.30; 0.63]	0.35 [0.25; 0.84]	
	**V2**	0.53 [0.33; 0.70]	0.43 [0.25; 0.97]	0.53 [0.36; 0.99]	0.64 [0.40; 1.21]	0.53 [0.30; 0.87]	0.53 [0.28; 0.99]	0.763
	**V3**	0.37 [0.21; 0.60]	0.31 [0.24; 1.00]	0.50 [0.25; 1.20]	0.42 [0.27; 1.29]	0.39 [0.29; 0.79]	0.38 [0.25; 0.85]	0.747

**ConA**								
**IFN-y (pg/ml)**	**V1**	34.70 [13.87; 111.50]	62.74 [11.32; 91.32]	48.22 [18.40; 111.62]	40.19 [10.72; 132.63]	32.48 [11.14; 130.03]	61.53 [11.13; 91.95]	
	**V2**	35.80 [9.58; 75.46]	22.23 [9.42; 63.45]	25.07 [10.10; 52.81]	41.07 [15.10; 108.30]	39.56 [8.74; 116.23]	20.80 [8.70; 78.25]	0.749
	**V3**	47.13 [12.50; 85.50]	32.54 [10.38; 82.70]	41.00 [16.71; 91.30]	50.04 [24.05; 119.46]	34.84 [10.38; 138.57]	30.78 [12.81; 74.70]	0.630
**TNF-α (pg/ml)**	**V1**	85.30 [7.97; 213.51]	70.86 [14.25; 155.38]	96.90 [38.92; 230.47]	60.76 [13.03; 156.52]	50.81 [9.51; 201.71]	81.21 [19.47; 259.56]	
	**V2**	28.39 [4.17; 177.95]	58.09 [23.96; 116.46]	43.05 [3.18; 120.88]	31.16 [5.60; 103.96]	27.67 [6.89; 177.09]	51.36 [4.19; 148.31]	0.499
	**V3**	25.71 [6.40; 123.18]	53.08 [10.63; 163.02]	30.74 [7.90; 132.23]	36.20 [9.59; 134.82]	33.54 [7.60; 165.18]	89.34 [9.68; 162.84]	0.214
**IL-1β (pg/ml)**	**V1**	2.77 [1.22; 7.08]	2.75 [1.05; 8.02]	2.76 [1.23; 9.10]	3.29 [0.98; 7.34]	3.39 [1.08; 7.73]	3.88 [1.98; 6.62]	
	**V2**	2.46 [0.70; 7.13]	2.05 [0.74; 5.60]	1.69 [0.60; 4.54]	3.35 [0.72; 9.35]	2.21 [1.13; 10.52]	2.44 [0.61; 5.82]	0.741
	**V3**	2.29 [1.54; 5.54]	2.57 [1.09; 7.32]	2.60 [1.74; 5.18]	3.09 [1.15; 9.01]	2.20 [0.74; 8.94]	3.39 [1.10; 7.70]	0.756
**IL-2 (pg/ml)**	**V1**	63.72 [26.96; 118.33]	60.60 [26.90; 111.86]	62.59 [28.94; 104.50]	62.32 [12.16; 125.84]	61.75 [24.24; 116.86]	72.19 [40.66; 107.62]	
	**V2**	34.04 [12.54; 74.87]	34.21 [14.92; 63.76]	45.39 [7.84; 76.78]	36.16 [9.13; 100.21]	49.96 [12.27; 85.09]	48.20 [8.34; 81.41]	0.384
	**V3**	43.45 [15.82; 78.50]	38.82 [21.82; 88.05]	56.24 [25.52; 90.80]	50.23 [18.32; 86.15]	48.92 [13.88; 72.20]	41.69 [17.80; 92.07]	0.570
**IL-12 (pg/ml)**	**V1**	0.98 [0.25; 2.42]	0.49 [0.25; 1.08]	0.28 [0.21; 1.79]	0.26 [0.25; 1.65]	0.35 [0.24; 1.12]	0.97 [0.25; 1.74]	
	**V2**	0.48 [0.25; 1.08]	0.29 [0.24; 0.68]	0.43 [0.25; 1.04]	1.01 [0.29; 1.28]	0.64 [0.29; 1.28]	0.68 [0.27; 1.28]	0.255
	**V3**	0.96 [0.24; 1.08]	1.02 [0.30; 1.08]	0.96 [0.23; 1.10]	1.02 [0.24; 1.09]	0.68 [0.25; 1.10]	0.68 [0.27; 1.05]	0.691
**IL-10 (pg/ml)**	**V1**	16.38 [10.00; 30.50]	15.71 [8.23; 25.39]	26.57 [10.14; 40.91]	15.94 [4.19; 36.63]	15.08 [8.12; 27.27]	20.05 [10.93; 32.91]	
	**V2**	13.95 [6.30; 29.14]	13.26 [6.49; 21.09]	16.48 [5.73; 32.10]	13.14 [4.62; 29.27]	12.96 [6.83; 30.69]	14.69 [2.34; 26.48]	0.366
	**V3**	13.69 [5.99; 19.63]	16.45 [9.35; 24.99]	23.38 [11.37; 36.69]	13.98 [7.90; 28.47]	11.18 [7.01; 25.93]	16.34 [7.05; 29.23]	0.384
**LPS**								
**IFN-y (pg/ml)**	**V1**	190.26 [100.53; 622.69]	127.44 [69.09; 344.51]	191.52 [120.31; 332.25]	148.97 [74.92; 365.55]	283.21 [125.36; 497.27]	211.94 [119.71; 513.05]	

	**V2**	219.68 [108.13; 386.41]	153.22 [53.79; 454.64]	135.07 [58.65; 256.39]	152.12 [56.39; 319.78]	243.46 [91.78; 485.87]	144.06 [51.82; 281.16]	0.599
	**V3**	207.58 [114.44; 478.65]	135.65 [53.97; 343.36]	122.12 [72.00; 300.76]	191.21 [99.52; 340.61]	223.50 [116.33; 729.92]	196.50 [75.60; 371.11]	0.479
**TNF-*α* (pg/ml)**	**V1**	1920.05 [875.35; 3192.41]	1585.50 [766.10; 2760.56]	1996.11 [937.91; 2776.47]	1288.74 [860.01; 2113.24]	2186.07 [1329.46; 2942.99]	1813.64 [1280.79; 3081.43]	
	**V2**	1733.72 [749.32; 2537.80]	1642.58 [790.33; 2525.10]	1586.06 [775.83; 2306.21]	950.42 [584.58; 2434.23]	1749.96 [752.07; 3215.91]	1628.76 [877.00; 2873.93]	0.597
	**V3**	1948.73 [1076.87; 2440.55]	1442.28 [638.99; 2036.82]	1223.17 [708.66; 2380.69]	1134.53 [575.88; 2291.24]	1762.24 [974.15; 3020.33]	1350.91 [790.52; 2453.38]	0.852
**IL-1β (pg/ml)**	**V1**	1508.49 [1009.93; 2594.92]	1251.46 [752.36; 2470.21]	1615.30 [1044.15; 3635.33]	1250.93 [868.42; 2288.95]	1637.93 [942.87; 2906.59]	1410.11 [1032.36; 3047.15]	
	**V2**	1643.08 [1077.03; 2160.01]	1468.94 [801.70; 2308.04]	1379.13 [825.53; 2451.17]	1476.79 [639.00; 2237.73]	1605.30 [897.62; 2818.60]	1407.76 [648.75; 2255.10]	0.513
	**V3**	1500.29 [925.36; 2918.14]	1488.54 [917.26; 2179.09]	1559.85 [1093.79; 2301.22]	1549.85 [741.79; 2279.49]	1569.56 [1068.69; 3419.61]	1533.39 [758.55; 2788.15]	0.947
**IL-2 (pg/ml)**	**V1**	0.24 [0.09; 1.16]	0.16 [0.08; 0.39]	0.10 [0.08; 0.45]	0.11 [0.10; 0.47]	0.12 [0.08; 0.39]	0.11 [0.08; 0.39]	
	**V2**	0.33 [0.11; 0.34]	0.30 [0.11; 0.33]	0.32 [0.11; 0.36]	0.33 [0.11; 0.36]	0.33 [0.16; 0.36]	0.33 [0.12; 0.34]	0.186
	**V3**	0.09 [0.08; 0.31]	0.10 [0.08; 0.34]	0.14 [0.08; 0.36]	0.09 [0.08; 0.34]	0.09 [0.07; 0.31]	0.24 [0.08; 0.32]	0.142
**IL-12 (pg/ml)**	**V1**	3.85 [1.03; 15.02]	1.58 [0.57; 5.99]	4.69 [1.14; 10.44]	2.74 [0.43; 8.96]	3.43 [1.69; 8.73]	3.35 [1.10; 8.75]	
	**V2**	4.50 [0.77; 14.08]	1.12 [0.27; 6.81]	1.04 [0.22; 6.30]	2.34 [0.48; 5.88]	2.33 [0.48; 8.36]	1.02 [0.27; 4.69]	0.125
	**V3**	3.78 [1.06; 7.54]	1.04 [0.60; 3.78]	1.52 [0.25; 6.59]	1.08 [0.83; 9.98]	3.46 [0.80; 5.95]	1.46 [0.71; 4.82]	0.259

	V1	129.15 [73.61; 202.73]	121.15 [83.41; 179.76]	156.10 [92.63; 229.86]	140.71 [88.87; 209.69]	120.48 [79.99; 197.14]	110.73 [63.24; 209.19]	
**IL-10 (pg/ml)**	V2	99.55 [54.16; 178.56]	137.89 [96.54; 185.09]	163.37 [66.42; 256.10]	126.93 [85.44; 162.26]	121.03 [60.25; 181.78]	126.64 [54.28; 173.87]	0.657
	V3	108.17 [59.11; 169.59]	107.66 [67.54; 211.88]	150.77 [73.92; 213.95]	114.12 [76.80; 176.79]	117.43 [84.39; 171.44]	105.15 [81.52; 165.19]	0.215

Values are presented as medians [Q1; Q3]. *P* represent the p-values for the differences over time (V1 to V2 and V1 to V3) between the six intervention groups tested with a Kruskal-Wallis test. * represents p<0.05 for the pairwise comparisons for differences between V1 and V3 compared to CTRL, tested by uncorrected Mann-Whitney U test. NPS: non-digestible polysaccharide, IFN: interferon, TNF: tumour necrosis factor, IL: interleukin, YBG: yeast β-glucan, SBG: shiitake β-glucan, OBG: oat β-glucan, AX: arabinoxylan, EPS: exopolysaccharide, CTRL: control.

Table S3. Faecal short-chain fatty acid levels and faecal pH at baseline (V1) and after five weeks of NPS consumption (V4)

		OBG	AX	CTRL	*P*
**Acetate (μmol/g)**	**V1**	40.57 [31.20; 60.23]	35.35 [18.76; 55.89]	32.02 [21.38; 42.66]	
	**V4**	35.52 [22.80; 48.17]	33.58 [22.90; 47.92]	34.37 [25.00; 53.36]	0.259
**Propionate (μmol/g)**	**V1**	11.17 [9.44; 15.93]	10.21 [6.38; 18.03]	10.38 [6.87; 13.43]	
	**V4**	9.49 [6.99; 16.26]	11.73 [7.95; 14.56]	10.97 [6.02; 15.24]	0.436
**Butyrate (μmol/g)**	**V1**	11.17 [7.56; 16.07]	7.58 [4.34; 16.93]	6.69 [4.76; 12.79]	
	**V4**	9.04 [6.00; 14.28]	8.27 [5.68; 12.65]	8.27 [4.57; 14.26]	0.129
**Total SCFA (μmol/g)**	**V1**	64.21 [46.13; 88.61]	51.95 [28.97; 89.44]	53.38 [33.45; 66.72]	
	**V4**	53.74 [37.55; 75.77]	53.57 [41.10; 86.22]	58.01 [36.48; 75.39]	0.210
**pH**	**V1**	7.56 [7.29; 7.70]	7.36 [7.10; 7.76]	7.48 [7.21; 7.76]	
	**V4**	7.50 [7.25; 7.78]	7.41 [7.12; 7.76]*	7.64 [7.22; 7.96]	0.018

Values are presented as medians [Q1; Q3]. *P* represent the p-values for the differences over time between the three intervention groups tested with a Kruskal-Wallis test. * represents p<0.05 for the pairwise comparisons for differences between V1 and V4 compared to CTRL, tested by uncorrected Mann-Whitney U test. NPS: non-digestible polysaccharide, OBG: oat β-glucan, AX: arabinoxylan, CTRL: control.

Table S4. Dietary intake at screening (V0) and after five weeks of NPS consumption (V4)

		YBG	SBG	OBG	AX	EPS	CTRL	*P*
**Energy (kJ/day)**	**V0**	9358.50 [7513.33; 11574.00]	8822.99 [6758.60; 10454.20]	8028.90 [6687.00; 10313.09]	10202.90 [8015.85; 11468.50]	8243.59 [6828.90; 10386.30]	9423.99 [7467.50; 11912.99]	
	**V4**	9079.55 [7553.68; 11366.53]	8787.09 [7120.20; 10643.59]	7993.70 [6247.60; 10025.09]	9278.09 [7401.75; 11944.60]	8073.80 [6253.20; 10246.20]	8565.59 [7150.90; 11053.80]	0.576
**Fibre (g/day)**	**V0**	21.38 [16.51; 26.13]	19.95 [16.45; 25.96]	17.99 [14.89; 26.33]	23.39 [17.76; 28.83]	18.72 [15.48; 22.70]	21.89 [17.75; 27.56]	
	**V4**	19.12 [14.72; 22.85]	18.46 [14.06; 24.06]	16.32 [12.73; 21.76]	19.54 [15.15; 25.84]	15.78 [13.35; 18.74]	18.99 [15.72; 24.18]	0.853
**Carbohydrates (g/day)**	**V0**	210.14 [170.12; 266.16]	209.54 [146.16; 245.38]	180.63 [153.30; 224.16]	221.05 [182.99; 265.24]	194.77 [146.53; 248.98]	206.28 [178.06; 271.78]	
	**V4**	223.11 [182.88; 275.49]	214.67 [166.01; 263.51}	206.68 [160.62; 239.89]	236.65 [174.29; 282.46]	203.64 [151.87; 243.92]	220.88 [171.55; 263.15]	0.555
**Fat (g/day)**	**V0**	101.43 [83.14; 121.72]	101.21 [79.42; 121.43]	86.65 [67.61; 119.02]	114.97 [89.03; 140.99]	98.53 [76.44; 114.77]	102.86 [77.03; 140.67]	

	V4	100.73 [74.47; 124.49]	93.83 [75.41; 115.24]	75.44 [63.30; 113.95]	103.01 [78.00; 122.84]	83.85 [58.42; 109.63]	94.21 [74.51; 117.94]	0.814
Protein (g/day)	V0	81.47 [63.92; 91.15]	71.20 [57.55; 8.17]	67.52 [54.17; 82.11]	81.65 [66.13; 104.70]	77.77 [58.74; 88.63]	80.42 [58.15; 113.22]	
	V4	75.62 [67.70; 94.44]	74.03 [51.82; 87.17]	62.91 [49.78; 85.41]	75.53 [56.72; 94.81]	68.90 [53.69; 82.40]	78.27 [54.45; 92.73]	0.386

Values are presented as medians [Q1; Q3]. *P* represent the p-values for the differences over time between the six intervention groups tested with a Kruskal-Wallis test. NPS: non-digestible polysaccharide, YBG: yeast β-glucan, SBG: shiitake β-glucan, OBG: oat β-glucan, AX: arabinoxylan, EPS: exopolysaccharide, CTRL: control.

Table S5. GI symptom scores, stool frequency and stool consistency at baseline (V1) and after five weeks of NPS consumption (V4)

		YBG	SBG	OBG	AX	EPS	CTRL	*P*
GI Symptom score	V1	16.06 [2.92; 45.44]	36.50 [15.33; 66.42]	31.39 [8.76; 60.58]	36.50 [10.58; 57.66]	59.85 [30.66; 86.13]	30.66 [5.11; 102.19]	
	V4	30.29 [6.93; 64.60]	56.93 [30.66; 82.48]	58.39 [23.36; 112.41]	59.12 [29.93; 88.32]	62.04 [33.58; 105.84]	37.96 [7.30; 92.70]	0.061
Max stool frequency	V1	7.00 [7.00; 7.00]	7.00 [6.00; 7.00]	7.00 [7.00; 14.00]	7.00 [5.00; 10.50]	7.00 [4.00; 14.00]	7.00 [7.00; 10.00]	
	V4	7.00 [7.00; 14.00]	7.00 [7.00; 7.00]	7.00 [7.00; 14.00]	7.00 [7.00; 14.00]	7.00 [5.00; 14.00]	7.00 [7.00; 14.00]	0.088
Min stool frequency	V1	7.00 [6.00; 7.00]	7.00 [5.00; 7.00]	7.00 [6.00; 7.00]	7.00 [5.00; 7.00]	7.00 [4.00; 7.00]	7.00 [6.00; 7.00]	

		YBG		SBG		OBG		AX		EPS		CTRL		P
	V4	7.00	[6.00; 7.00]	7.00	[5.00; 7.00]	7.00	[5.00; 7.00]	7.00	[6.00; 7.00]	7.00	[5.00; 7.00]	7.00	[7.00; 7.00]	0.534
Stool consistency	V1	1.00	[0.00; 3.00]	1.00	[0.00; 3.00]	0.50	[0.00; 3.00]	1.00	[-0.50; 2.00]	1.00	[0.00; 2.00]	1.00	[0.00; 2.00]	
	V4	1.00	[-0.25; 3.00]*	1.00	[0.00; 2.00]*	0.00	[0.00; 2.00]#	1.00	[-1.00; 3.00]*	1.00	[0.00; 3.00]	1.00	[0.00; 4.00]	0.029

Values are presented as medians [Q1; Q3]. *P* represent the p-values for the differences over time between the six intervention groups tested with a Kruskal-Wallis test. * represents $p<0.05$ for the pairwise comparisons for differences between V1 and V4 compared to CTRL, tested by uncorrected Mann-Whitney U test. # represents a $p<0.1$ for the pairwise comparisons for differences between V1 and V4 compared to CTRL, tested by uncorrected Mann-Whitney U test. GI: gastrointestinal, NPS: non-digestible polysaccharide, YBG: yeast β-glucan, SBG: shiitake β-glucan, OBG: oat β-glucan, AX: arabinoxylan, EPS: exopolysaccharide.

Table S6. Laboratory safety parameters at screening (V0) and after five weeks of NPS consumption (V4)

		YBG		SBG		OBG		AX		EPS		CTRL		*P*
Leucocytes (/nl)	V0	5.95	[4.90; 6.73]	6.30	[5.50; 7.10]	6.10	[5.40; 7.40]	6.10	[5.10; 7.05]	6.40	[5.70; 8.40]	6.40	[5.60; 7.05]	
	V4	5.00	[4.58; 5.83]	6.10	[5.00; 6.90]	5.50	[4.70; 6.20]	5.40	[4.35; 6.35]	5.60	[4.90; 7.00]	5.65	[4.78; 6.68]	0.338
Erythrocytes (/nl)	V0	4.55	[4.30; 4.70]	4.70	[4.40; 4.90]	4.60	[4.30; 4.90]	4.60	[4.20; 4.80]	4.50	[4.30; 4.80]	4.50	[4.40; 4.80]	
	V4	4.55	[4.38; 4.80]	4.70	[4.40; 5.00]	4.60	[4.40; 4.80]	4.50	[4.25; 4.90]	4.60	[4.40; 4.90]	4.70	[4.40; 4.83]	0.700
HB (g/dl)	V0	13.70	[13.18; 14.28	14.10	[13.40; 14.70]	14.00	[13.40; 14.40]	13.80	[12.70; 14.50]	13.90	[13.20; 14.40]	14.00	[13.20; 14.70]	

	**V4**	13.80 [13.30; 14.73]	14.20 [13.40; 15.00]	14.00 [13.50; 14.50]	13.80 [13.20; 14.55]	14.20 [13.30; 15.00]	14.05 [13.38; 14.90]	0.527
**Haematocrit (%)**	**V0**	41.35 [40.10; 43.38]	42.20 [40.60; 44.50]	42.50 [39.80; 44.60]	41.80 [39.55; 43.90]	42.20 [40.20; 43.50]	42.00 [40.28; 44.08]	
	**V4**	41.35 [39.95; 43.43]	43.00 [40.30; 44.90]	41.80 [40.90; 43.80]	41.50 [39.60; 44.10]	42.60 [40.20; 45.60]	42.35 [40.38; 44.68]	0.692
**MCV (fl)**	**V0**	92.90 [90.78; 95.13]	91.40 [88.60; 94.60]	92.00 [89.70; 93.60]	91.30 [89.90; 94.55]	91.70 [89.80; 93.60]	92.10 [90.10; 94.68]	
	**V4**	92.40 [89.75; 94.53]	91.60 [88.00; 94.00]	91.40 [89.10; 93.10]	90.00 [88.90; 93.90]	91.60 [89.20; 92.80]	91.50 [88.85; 94.28]	0.084
**MCH (pg)**	**V0**	30.80 [30.03; 31.53]	30.20 [29.00; 31.40]	30.30 [29.50; 31.20]	29.80 [29.25; 30.95]	30.20 [29.50; 31.20]	30.20 [29.60; 31.15]	
	**V4**	30.80 [29.80; 31.50]	30.30 [29.40; 31.30]	30.30 [29.80; 31.00]	30.20 [29.15; 31.55]	30.30 [29.60; 31.10]	30.50 [29.30; 31.55]	0.433
**MCHC (g/dl)**	**V0**	33.15 [32.60; 33.50]	32.90 [32.70; 33.30]	33.00 [32.70; 33.50]	32.80 [32.50; 33.10]	33.00 [32.80; 33.30]	32.90 [32.50; 33.35]	
	**V4**	33.30 [33.08; 33.63]	33.20 [32.80; 33.40]	33.20 [32.90; 33.50]	33.40 [33.05; 33.70]	33.10 [32.90; 33.50]	33.15 [32.80; 33.70]	0.079
**Thrombocytes (/nl)**	**V0**	266.00 [224.00; 294.25]	287.00 [223.00; 305.00]	281.00 [224.00; 313.00]	266.00 [245.00; 300.50]	261.00 [235.00; 308.00]	271.50 [218.50; 316.25]	

	**V4**	262.50 [219.50; 292.00]	270.00 [238.00; 300.00]	269.00 [237.00; 320.00]	281.00 [254.50; 315.00]	279.00 [238.00; 315.00]	262.50 [217.00; 314.50]	0.354
**MPV (fl)**	**V0**	7.80 [7.28; 8.58]	8.00 [7.30; 8.60]	7.70 [7.20; 8.50]	7.80 [7.35; 8.60]	8.10 [7.38; 8.53]	8.05 [7.60; 9.03]	
	**V4**	7.95 [7.20; 8.73]	8.10 [7.00; 8.80]	7.80 [7.10; 8.60]	7.80 [7.45; 8.55]	8.05 [7.50; 8.63]	8.20 [7.68; 9.23]	0.978
**Alkaline Phosphatase (U/l)**	**V0**	72.00 [61.50; 85.25]	83.00 [71.00; 90.00]	75.00 [61.00; 83.00]	74.00 [66.00; 89.50]	73.00 [62.00; 84.00]	69.00 [58.75; 80.25]	
	**V4**	74.50 [58.75; 89.00]	82.00 [71.00; 92.00]	73.00 [66.00; 87.00]	78.00 [66.00; 87.00]	73.00 [63.00; 91.00]	73.50 [65.00; 85.25]	0.513
**AST (U/l)**	**V0**	27.00 [24.00; 32.00]	27.00 [24.00; 33.00]	25.00 [23.00; 30.00]	28.00 [22.50; 31.50]	27.00 [23.00; 33.00]	27.00 [23.00; 35.25]	
	**V4**	26.50 [24.00; 32.25]	25.00 [22.00; 28.00]	26.00 [23.00; 29.00]	26.00 [23.00; 30.00]	26.00 [22.00; 32.00]	26.00 [23.00; 32.25]	0.103
**ALT (U/l)**	**V0**	33.00 [26.75; 39.00]	33.00 [27.00; 37.00]	31.00 [25.00; 40.00]	29.00 [24.50; 36.00]	30.00 [27.00; 37.00]	30.00 [28.00; 36.50]	
	**V4**	32.00 [29.00; 38.00]	33.00 [27.00; 36.00]	32.00 [27.00; 38.00]	31.00 [26.00; 37.00]	32.00 [27.00; 38.00]	33.50 [30.75; 39.00]	0.629
**GGT (U/l)**	**V0**	24.50 [18.00; 29.00]	24.00 [16.00; 34.00]	24.00 [20.00; 36.00]	28.00 [20.50; 35.50]	24.00 [19.00; 31.00]	27.00 [19.00; 40.50]	

	**V4**	25.50 [18.00; 33.00]	24.00 [16.00; 30.00]	25.00 [16.00; 30.00]	27.00 [21.50; 38.50]	26.00 [19.00; 31.00]	27.00 [20.00; 40.50]	0.519
**Cholinesterase (U/l)**	**V0**	7998.00 [6936.25; 9159.50]	8639.00 [7458.00; 9376.00]	8016.00 [7036.00; 9089.00]	8365.00 [7663.50; 9351.00]	8605.00 [7636.00; 9420.00]	8190.50 [7250.50; 8945.25]	
	**V4**	8054.50 [6790.50; 8788.75]	8671.00 [7691.00; 9356.00]	7816.00 [7058.00; 8963.00]	8252.00 [7293.50; 9253.50]	8432.00 [7293.50; 9253.50]	8173.00 [7208.25; 9290.50]	0.122
**Creatinine (mg/dl)**	**V0**	0.90 [0.78; 1.00]	0.90 [0.80; 1.00]	0.80 [0.70; 0.90]	0.90 [0.80; 1.00]	0.80 [0.70; 0.90]	0.90 [0.70; 1.00]	
	**V4**	0.90 [0.80; 1.00]	0.90 [0.80; 1.00]	0.80 [0.70; 0.90]	0.90 [0.80; 1.00]	0.80 [0.70; 0.90]	0.90 [0.80; 1.00]	0.922
**MDRD (ml/Min.)**	**V0**	74.00 [69.00; 85.25]	79.00 [66.00; 85.00]	83.00 [72.00; 95.00]	74.00 [65.00; 85.50]	84.00 [77.00; 95.00]	82.50 [73.75; 95.25]	
	**V4**	75.50 [71.00; 84.50]	74.00 [63.00; 85.00]	83.00 [73.00; 96.00]	75.00 [67.00; 84.00]	83.00 [73.00; 87.00]	83.00 [73.75; 95.00]	0.895
**Potassium (mmol/l)**	**V0**	4.60 [4.30; 4.80]	4.80 [4.40; 4.90]	4.70 [4.50; 4.90]	4.60 [4.40; 4.80]	4.60 [4.40; 4.80]	4.60 [4.30; 4.83]	
	**V4**	4.40 [4.18; 4.70]	4.40 [4.20; 4.60]	4.40 [4.30; 4.60]	4.40 [4.10; 4.60]	4.50 [4.30; 4.70]	4.50 [4.20; 4.80]	0.107

**Sodium (mmol/l)**	**V0**	142.00 [139.00; 143.00]	141.00 [140.00; 143.00]	141.00 [139.00; 142.00]	141.00 [139.50; 143.00]	141.00 [139.00; 143.00]	141.00 [139.00; 143.00]	
	**V4**	140.00 [140.00; 142.00]	142.00 [140.00; 142.00]	141.00 [139.00; 142.00]	141.00 [139.50; 143.00]	142.0 [140.00; 143.00]	140.50 [139.00; 143.00]	0.777
**Bilirubin (mg/dl)**	**V0**	0.75 [0.60; 1.03]	0.70 [0.50; 0.80]	0.80 [0.60; 0.95]	0.80 [0.60; 0.95]	0.80 [0.50; 0.90]	0.70 [0.50; 0.93]	
	**V4**	0.70 [0.60; 1.03]	0.70 [0.50; 0.90]	0.70 [0.60; 0.90]	0.70 [0.60; 0.90]	0.70 [0.50; 0.90]	0.85 [0.70; 1.10]	0.264

Values are presented as medians [Q1; Q3]. *P* represent the p-values for the differences over time between the six intervention groups tested with a Kruskal-Wallis test. NPS: non-digestible polysaccharide, YBG: yeast β-glucan, SBG: shiitake β-glucan, OBG: oat β-glucan, AX: arabinoxylan, EPS: exopolysaccharide, CTRL: control, HB: haemoglobin, MCV: mean corpuscular volume, MCH: mean corpuscular haemoglobin, MCHC: mean corpuscular haemoglobin concentration, MPV: mean platelet volume, AST: aspartate transaminase, ALT: alanine transaminase, GGT: gamma-glutamyl transferase, MDRD: modification of diet in renal disease.
